# Structure based function-annotation of hypothetical protein MGG_01005 from *Magnaporthe oryzae* reveals it is the dynein light chain orthologue of dynlt1/3

**DOI:** 10.1038/s41598-018-21667-5

**Published:** 2018-03-02

**Authors:** Guorui Li, Jinguang Huang, Jun Yang, Dan He, Chao Wang, Xiaoxuan Qi, Ian A. Taylor, Junfeng Liu, You-Liang Peng

**Affiliations:** 1 MOA Key Laboratory of Plant Pathology, China Agricultural University, No2 Yunamingyuanxilu, Beijing, 100193 China; 2 State key Laboratory of Agrobiotechnology, China Agricultural University, No2 Yunamingyuanxilu, Beijing, 100193 China; 3 College of life science, Inner Mongolia University for Nationalities, No. 996 Xilamulun Street, Tongliao, 028043 China; 4 College of Agronomy and Plant Protection, Qingdao Agricultural University, Qingdao, Shandong 266109 China; 5 Macromolecular Structure Laboratory, The Francis Crick Institute, London, NW1 1AT UK

## Abstract

*Magnaporthe oryzae* is a model fungal plant pathogen employed for studying plant-fungi interactions. Whole genome sequencing and bioinformatics analyses revealed that this fungal pathogen has more than 12,000 protein-coding genes with 65% of the genes remaining functionally un-annotated. Here, we determine the structure of the hypothetical protein, MGG_01005 and show that it is the *Magnaporthe oryzae* Dynein light chain Tctex-type 1 (dynlt1/3), demonstrated by its structural similarity to other orthologous dynlt1 proteins and its conserved interaction with the N-terminus of the *Magnaporthe oryzae* dynein intermediate chain, MoDyn1I2. In addition, we present the structure of the MGG_01005-MoDyn1I2 complex together with mutagenesis studies that reveals a di-histidine motif interaction with a glutamate residue in the dynein intermediate chain within a conserved molecular interface. These results demonstrate the utility of structure-based annotation and validate it as a viable approach for the molecular assignment of hypothetic proteins from phyto-pathogenic fungi.

## Introduction

Rice blast fungus (*Magnaporthe oryzae*) is the pathogen that causes rice blast disease, one of the most devastating diseases to affect rice production^1,2^. It is widely recognized as a model organism to study the molecular mechanisms of fungal pathogenesis^3–5^. Elucidating the mechanism for growth, development and pathogenicity of the fungus will aid and facilitate improved ways to control rice blast disease. The genome of *M. oryzae* was the first plant pathogenic fungus to be sequenced^6^. Subsequently, the genomes of more than 4 other strains or isolates of *M. oryzae* and two other species of the *Magnaporthaceae* family have been sequenced^7–9^. However, despite the overwhelming impact of genomic sequencing on the understanding the biology and evolution of the fungi, the gap between gene sequence and protein function remains in a significant proportion of cases.

The traditional way to annotate and ascribe function to a hypothetic protein is to compare the amino acid sequence of the protein against all functionally assigned sequences in protein sequence databases. If there is significant sequence or motif identity between the protein and a functionally assigned protein, then it is predicted that these two proteins share similar functions. However, many of the newly generated protein sequences cannot be assigned with precise function based on this strategy. Therefore, because these hypothetic or uncharacterised proteins may be important to the life cycle and pathogenicity of the fungus, a major challenge is to find ways to reliably and rapidly predict or determine the molecular functions of these proteins.

One other approach to assign the molecular function of a protein is to compare its three-dimensional structure determined by either X-ray crystallography or solution state NMR against those of the protein structure database (PDB: Protein Data Bank). If one or more significant structural homologues have been deposited in PDB, the uncharacterised protein is predicted to have similar molecular properties to the homologues. These predictions can then be tested by other experiments such as binding or activity analysis. This three-dimensional structure comparison is far more sensitive than primary sequence comparisons because proteins which adopt similar tertiary structures often share similar molecular functions although they may not have significant sequence similarity^10^.

Here we present the crystal structure of the protein encoded by *MGG_01005*, a gene that is important for vegetative mycelial growth of *M. oryzae*^11^. Although there is only low sequence conservation with the Dynein light chain Tctex-type 1 (dynlt1/3), from yeast, human, mouse and fruit fly^12–14^, the crystal structure of the gene product, MGG_01005, adopts the dynein light-chain Tctex-1 fold. Pull-down and yeast two-hybrid analysis demonstrate an interaction with the N-terminal region of the *M. oryzae* dynein intermediate chain (DIC) MoDyn1I2 as observed for other dynlt1/3 proteins confirming MGG_01005 as the dynlt1/3 of *M. oryzae*. Importantly, the structure of a complex of MGG_01005 with an N-terminal peptide from MoDyn1I2^117–150^ together with mutagenesis studies reveal that a di-histidine motif, widely conserved in dynlt1/3 proteins interacts with a highly conserved glutamate residue in the DIC. These results highlight how 3-D structure determination may suggest further biophysical or biochemical experiments that allow the determination the molecular functions of predicted proteins associated with vegetative growth, asexual development or pathogenicity in Rice blast fungi.

## Results

### Structure of MGG_01005

**Figure 1:**
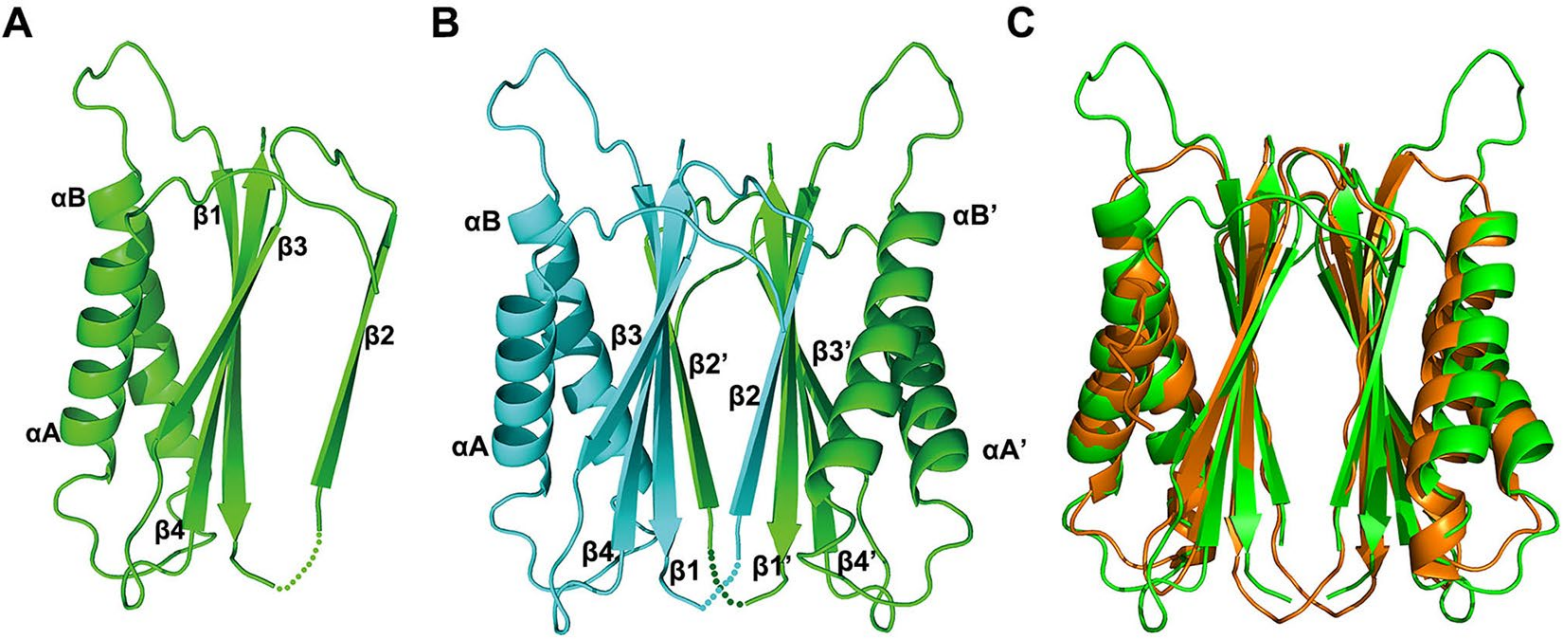
Structure of MGG_01005. (**A**) Structure of MGG_01005. The structure is shown in cartoon representation, α-helices and β-strands are indicated. (**B**) The MGG_01005 dimer, Chain A and B are coloured green and cyan, respectively. The secondary structure elements are labelled as in (**A**). (**C**) Superposition of MGG_01005 (green) and *D. melanogaster* dynlt1/3 (1YGT, orange).

The 1.9 Å structure of the MGG_01005 was solved by single-wavelength anomalous diffraction (SAD). Details of the data collection and refinement statistics are presented in Table 1. There is one protein molecule in the asymmetric unit (ASU) and the final refined model (Fig. 1A) contains all residues except those between 80 and 112 that are not visible in the electron density map and are likely to be flexible or disordered. In addition, electron density for three extra residues (HHS) from the N-terminal His-tag is present (Supplementary Figure S1**)**. The monomer structure consists of two α helices (A to B) and 4 β-strands (β1-4). Three β-strands (β1-β3-β4) comprise an antiparallel β-sheet. The fourth strand β2, packs against the equivalent sheet (β1′-β3′-β4′) of a symmetry related molecule to form a dimer comprising an 8 stranded β-sandwich structure where each monomer has the topological arrangement αA-αB-β1-(β2′)-β3-β4, Fig. 1B. The configuration of this strand-swapped dimer and the magnitude of buried surface area: 1654 Å^2^ comprising 154 atoms from 43 residues in each molecule and a homodimer interface stabilized by 36 hydrogen bonds and 2 salt bridges (Supplementary Figure S2) suggests strongly that MGG_01005 is homodimeric in solution. This observation was confirmed by gel filtration chromatography and sedimentation velocity analytical ultracentrifugation measurements that demonstrate the dimer mass in solution (Supplementary Figure S3). A large proportion of the homodimeric interface is formed by the two layers of the anti-parallel β-sandwich (Fig. 1B) and more than twenty hydrophobic interactions and fifteen hydrogen bonding interactions between residues in the sandwich contribute to stabilisation of the homodimer (Supplementary Figure S2A,B). A similar pattern of hydrogen bonding is also observed in the *Dm* dynlt1/3 homodimer interface (Supplementary Figure S2C,D). Among these interactions, Lys68 on β1 of one monomer (Monomer-A) interacts with Asp127′ on β3′ of the other monomer (Monomer-B) through two salt bridges. In addition to interactions at the sandwich interface, there are also further hydrogen bonding interactions between Asn41 in αB of monomer-A and His115′ in β1′ in Monomer-B (Supplementary Figure S2B).

**Table 1:** X-ray data collection and refinement statistics.

	MGG_1005	MGG_1005-IC^117–150^
Wavelength (Å)	0.9791	0.9793
Resolution range (Å)	46.53–1.97 ^†^(2.041–1.97)	29.63–2.4 (2.486–2.4)
Space group	P 3 2 1	I 41 2 2
Unit cell	72.64 72.64 46.53 90 90 120	105.097 105.097 166.541 90 90 90
Total reflections	86058 (3961)	463744 (44369)
Unique reflections	10135 (644)	18442 (1811)
Multiplicity	8.5 (6.2)	25.1(24.5)
Completeness (%)	98.5(90.8)	99.04 (98.96)
Mean I/sigma(I)	17.3 (5.1)	28.0(2.6)
Wilson B-factor	19.94	48.33
R-merge	0.081 (0.408)	0.038(0.477)
CC_1/2_	0.999 (0.888)	0.998(0.812)
CC*	1 (0.97)	0.999(0.905)
Reflections used for R-free	1019	1844
R-work	0.1720 (0.1879)	0.2057 (0.2775)
R-free	0.2074 (0.2448)	0.2350 (0.3224)
CC(work)	0.947(0.909)	0.943 (0.773)
CC(free)	0.918(0.847)	0.910 (0.755)
Number of non-hydrogen atoms	1014	2223
macromolecules	960	2147
ligands		
water	54	76
Protein residues	123	277
RMS(bonds)	0.008	0.010
RMS(angles)	1.02	1.19
Ramachandran favored (%)	98	99
Ramachandran allowed (%)	2	1
Ramachandran outliers (%)	0	0
Clashscore	3.73	6.61
Average B-factor	30.40	55.30
macromolecules	30.00	55.80
ligands	/	/
solvent	38.30	44.00

^†^Values for the highest-resolution shell are shown in parentheses.

### Structural comparison with dynein light chain proteins

Structural similarity searches of the PDB with MGG_01005 using the SSM server^15^ produced the strongest matches with the dynlt1/3 proteins, from *Drosophila melanogaster* (*Dm* dynlt1; 1YGT, Q score = 0.609; 3FM7, Q score = 0.528) and *Chlamydomonas reinhardtii* (*Cr* dynlt1; 1XDX, Q score = 0.393) (Supplementary Table S1). The best-fit Cα superposition of MGG_01005 with *Dm* dynlt1/3 is shown in Fig. 1C. The rmsd and sequence similarity identity compared with *Dm* dynlt1/3 and *Cm* dynlt1/3 are 1.29 Å, 17.9% and 2.33 Å, 19.5% respectively. Inspection of the *Dm* dynlt1/3 structural superposition reveals that the six secondary structural elements of a MGG_01005 monomer superpose very well with those of *Dm* dynlt1, Fig. 1C. The only significant deviations are in the loop connecting αB and β1 in MGG_01005 that is eight residues longer than that of *Dm* dynlt1/3. Additionally, residues 80 to 112, that are not visible in the model although conserved in the MGG_01005 orthologues of some fungi, are missing in the orthologues of yeast, mammals and fruit fly (Fig. 1C, Supplementary S4)^12,13,16,17^. Inspection of structure-based sequence alignments with other dynlt1/3 family members (Supplementary Figure S4**)** reveals the conservation of secondary structure elements. Although, notably the secondary structures of MGG_01005 are longer than those of other dynlt1/3 family members. Nevertheless, taken together, these data strongly support that MGG_01005 is the *Magnaporthe oryzae* orthologue of dynlt1/3.

### Interaction of MGG_01005 and dynein intermediate chain

Dynlt1/3, like other dynein light chain family members is often linked to dynein heavy chains through interaction with a dynein intermediate chain (DICs)^18,19^. Therefore, the interaction between MGG_01005 and *M. oryzae* DICs was analysed by co-immunoprecipitation coupled with mass spectrometry, yeast two-hybrid assay (Y2H) and isothermal titration Calorimetry (ITC). To identify MGG_01005 associated proteins in *M. oryzae* a transformant, PDTC1, that expressed an MGG_01005-3XFlag fusion protein was generated in an MGG_01005 deletion strain. A 23 kDa band of the expected size of MGG_01005-3X Flag was detected with an anti-Flag antibody in the proteins isolated from vegetative hyphae of PDTC1^11^. To identify proteins that interact with MGG_01005, total proteins isolated from the PDTC1 strain were immunoprecipitated with anti-FLAG M2 beads and the co-immunoprecipitated proteins digested with trypsin and the peptides analysed by LC-MS/MS. The resulting proteins identified from this procedure are listed in Supplementary Table S2. Notably, along with MGG_01005 itself, MGG_04771, which encodes the cytoplasmic dynein 1 intermediate chain 2 (MoDyn1I2), was identified as a top hit.

**Figure 2:**
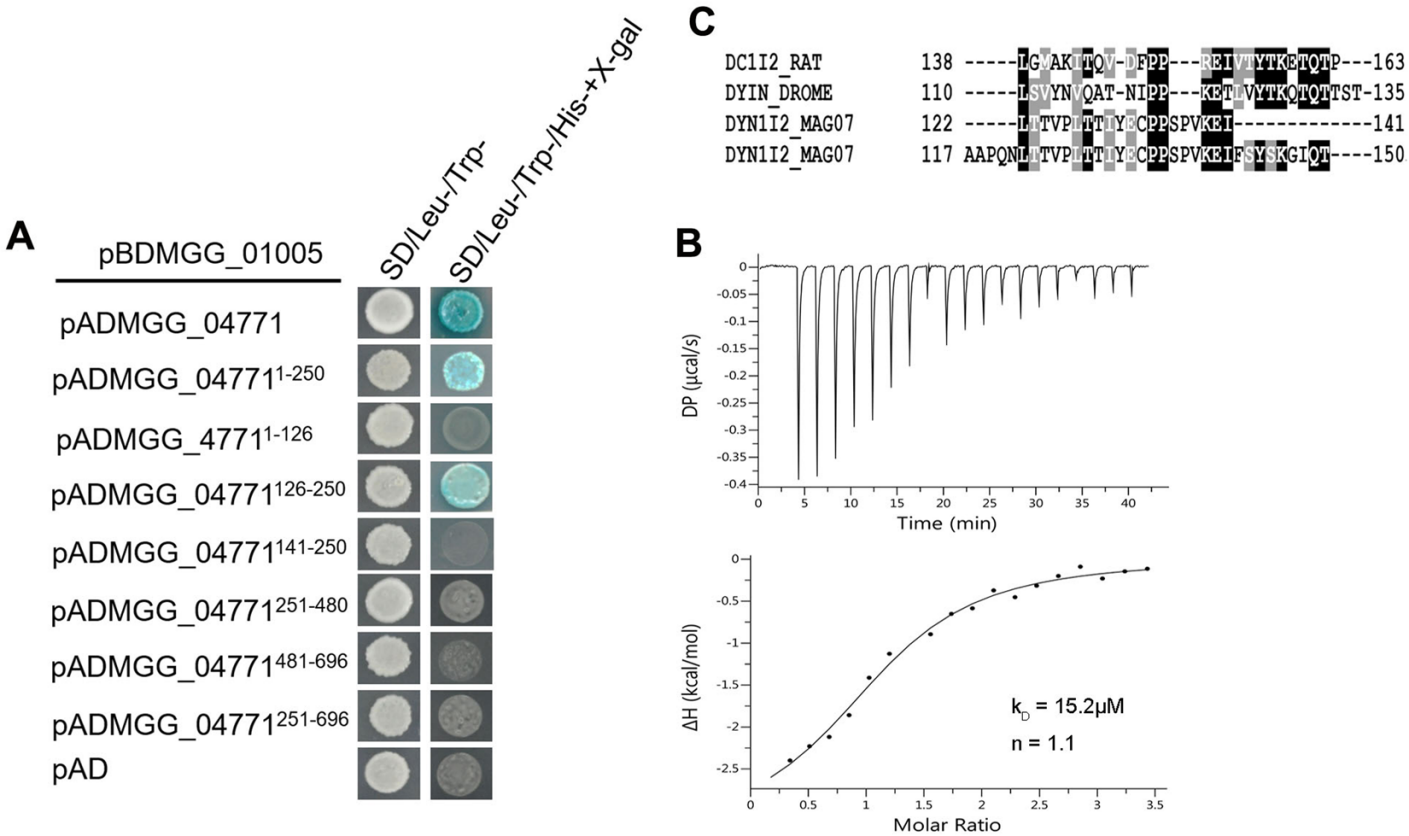
MGG_01005 - dynein intermediate chain interaction. (**A**) Yeast two-hybrid analysis of interactions between MGG_01005 and full-length and deletion mutants of MGG_04771 (MoDyn1I2). (**B**) ITC thermogram for MGG_01005 binding to a MoDyn1I2^(122–143^) peptide. A representative experiment from three different repeats is shown (K_D_ = 15.2 μM ± 5.97 μM, n = 1.13 ± 0.05, ∆H (kcal/mol) = −3.28 ± 0.406, ∆G (kcal/mol) = −6.58). (**C**) Primary sequence alignment of the dynein intermediate chain peptide (ICp) dynlt1/3 binding motif. Highly conserved residues are indicated with a black background.

To further investigate whether MGG_01005 directly interacts with MoDyn1I2, the interaction was analysed using yeast a two-hybrid (Y2H) approach. Here, a MGG_01005 bait construct was co-transformed with a MoDyn1I2 prey construct into yeast strain AH109. The resulting Trp^+^ Leu^+^ transformants were able to grow on SD-His medium and display LacZ activities (Fig. 2A), suggesting MGG_01005 directly interacts with MoDyn1I2 in yeast cells. To more finely map which region was responsible for the interaction, a deletion series of MoDyn1I2 prey constructs was tested, and residues 126–250 of MoDyn1I2 (MoDyn1I2^126–250^) were found to be sufficient for the interaction with MGG_01005 (Fig. 2A). Moreover, a shorter construct comprising residues 142–250 of MoDyn1I2 (MoDyn1I2^142–250^) did not interact with MGG_01005, suggesting that the region containing residues 126–141 was indispensable for the interaction between MoDyn1I2 and MGG_01005.

To further characterize the MGG_01005 and MoDyn1I2 interaction, the binding of MGG_01005 with a MoDyn1I2 derived peptide, residues 122–141 was measured by ITC (Fig. 2B). These data reveal binding of the MoDyn1I2^122–141^ peptide to MGG_01005 with an equilibrium dissociation constant of 15.2 μM ± 5.97 μM and n = 1.1 and favourable enthalpic component ∆H (kcal/mol) = −3.28 ± 0.406 clearly demonstrating that MGG_01005 interacts with the N-terminal region of the dynein intermediate chain, MoDyn1I2 and that two MoDyn1I2^122–141^ peptides can interact with the two identical interfaces in the MGG_01005 dimer.

### Structure of MGG_01005-MoDyn1I2 peptide complex

**Figure 3:**
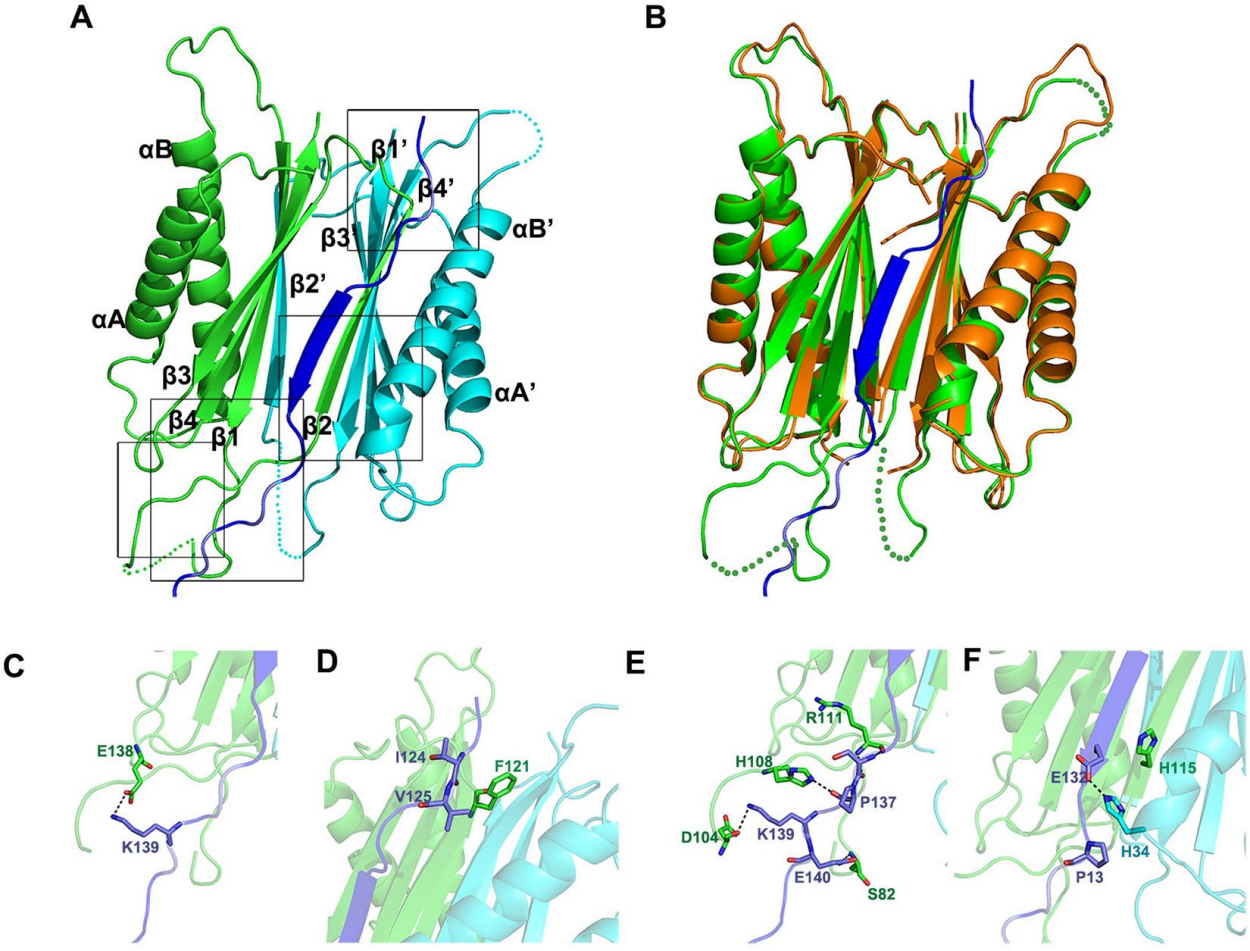
Crystal structure of MGG_01005-MoDyn1I2^117–150^ complex. (**A**) The structure of the MGG_01005-MoDyn1I2^117–150^ complex shown in cartoon representation. Chain A of MGG_01005 is shown in green, Chain B in cyan and MoDyn1I2^117–150^ in blue. The dashed lines indicate missing residues, 85–103 and 59–62. (**B**) Superposition of MGG_01005 (orange) and MGG_01005- MoDyn1I2^117–150^ complex (green and blue). (**C**–**F**) Close up views of MGG_01005- MoDyn1I2^117–150^ interactions, hydrogen bonds are shown as dashed lines: (**C**) E138 of MGG_01005 and K139 of MoDyn1I2^117–150^, (**D**) F121 of MGG_01005 and I124-V125 of MoDyn1I2^117–150^, (**E**) N and C-terminal residues of the 80–112 loop of MGG_01005 and P137-E140 of MoDyn1I2^117–150^, (**F**) H34 and H115 of MGG_01005 and E132 of MoDyn1I2^117–150^.

To determine the structure and gain insight into the molecular details of the MGG_01005 - MoDyn1I2 interaction, we attempted to co-crystallise MGG_01005 with a variety MoDyn1I2 derived peptides. Using this approach, and the apo-MGG_01005 structure as a search model, we were successful in solving an MGG_01005 - MoDyn1I2^117–150^ peptide complex structure by molecular replacement using PHASER implemented in the PHENIX platform (McCoy *et al*.^36^, Adams *et al*.^38^). Data collection and refinement statistics are presented in Table 1. In the complex, there are two copies of MGG_01005 and one MoDyn1I2^117–150^ peptide per asymmetric unit (Fig. 3A). No electron density was observed for MGG_01005 residues Chain A 1–4, and 85–103 and Chain B 102, 59–62 and 81–112 and residues 117–122 and 150 of MoDyn1I2^117–150^, indicating that these regions are likely disordered in the crystal.

Structural superposition of the apo and MGG_01005 – peptide complex reveals a high degree of similarity. The rmsd after alignment of Cα positions of the A chains is 0.7 Å (Fig. 3B) indicating that there is no large conformational change in MGG_01005 upon binding to the MoDyn1I2^117–150^ peptide. Notably, residues of 80–84 and 104–112, which could not be built in the model of apo structure, are present in chain A of the complex. By comparison, there is no electron density for residues of 59–62 of chain B of the complex although these residues are visible in the apo structure. These changes likely result from stabilisation by the interaction with the peptide in the complex or destabilisation through loss of contacts with the symmetry molecule in the apo structure.

Notably, two MGG_01005-MoDyn1I2^117–150^ interfaces are observed in the crystal structure of the complex. In the first, residues of 122–141 of the MoDyn1I2^117–150^ peptide interact with a single MGG_01005 dimer within the ASU (Fig. 3). In addition, residues 142–150 at the C-terminus of MoDyn1I2^117–150^ also extend from one MGG_01005 dimer (Supplementary Figure S5A) and pack onto a symmetry-related MGG_01005 dimer resulting in an arrangement in the crystal where 3 peptides saturate 4 binding sites on 2 adjacent dynlt1/3 dimers (Supplementary Figure S5B). However, it is likely that this second interface comprising the residue 142–150 interaction with the symmetry related molecule (Supplementary Figure S5C,D) is not biologically relevant and is due to the high protein concentration in the crystal as these C-terminal residues were dispensable for the interaction with MGG_01005 in ITC binding assays (Fig. 2B) and none of the residues in this region were essential for the interaction with MGG_01005 in the Y2H analysis (Supplementary Figure S6). Notably, in addition non-unitary stoichiometries (0.4 to 0.7) are observed in ITC titrations that employ the long MGG_01005-MoDyn1I2^117–150^ peptide (Supplementary Figure S5E), possibly as a result of a mixed mode of binding that observed in the crystal structure. By comparison the expected stoichiometry of 1 peptide per MGG_01005 monomer was consistently observed for the MGG_01005-MoDyn1I2^122–141^ interaction. Therefore, based on these data, the MoDyn1I2^122–141^- MGG_01005 dimer interface appears to be the most biologically relevant and so was further analysed in detail.

### MGG_01005- MoDyn1I2 interactions

The MGG_01005-MoDyn1I2 interface buries 2811 Å^2^ of the protein surface. This combines 2157 Å^2^ from monomer A of MGG_01005 and 654 Å^2^ monomer B. At the interface, 30 residues of the MGG_01005 homodimer interact with 18 residues (123′−141′ excepting 127′) of the MoDyn1I2 peptide. In agreement with previous models of dynlt1/3-DIC complexes^14^ the structure shows that residues 122–141 of MoDyn1I2 bind to the edge of the MGG_01005 β-sandwich and contribute an additional strand to the antiparallel β-sheet (Fig. 3A). However, additionally in the MGG_01005-MoDyn1I2 complex residues in the MGG_01005 flexible region, residues 80–112, that are only present in the fungal orthologues, make further interactions with MoDyn1I2.

Inspection of the protein-protein interface reveals that MGG_01005 mainly makes hydrophobic interactions and mainchain - mainchain hydrogen bonds with the MoDyn1I2^117–150^ peptide (Supplementary Figure S7A,B). This includes 25 hydrophobic interactions and 16 hydrogen bonds in the interface between MGG_01005 monomer A and MoDyn1I2^117–150^. Among these interactions, E138 makes a salt bridge with K139 of MoDyn1I2^117–150^ and the F121 side chain makes hydrophobic interactions with both the T124 main chain and V125 side chain of MoDyn1I2^117–150^ (Fig. 3C,D). In addition, residues at the N- and C-termini of the 80–112 loop, which is only present in the orthologues of fungal species, interact with the MoDyn1I2^117–150^ through 10 hydrophobic interactions and 6 hydrogen bonds **(**Fig. 3E**)**. At the interface with MGG_01005 Monomer B there are seven hydrophobic interactions and one salt bridge, largely mediated by H34 and a conserved EXPP motif comprising residues 132–135 of MoDyn1I2^117–150^ (Fig. 2C). Here, H34 makes both a salt bridge and a hydrophobic interaction with E132 of the motif (Fig. 3F) whilst additionally the aliphatic portion of the chain E132 of MoDyn1I2^117–150^ also interacts with H115 of monomer A by a hydrophobic interaction.

**Figure 4:**
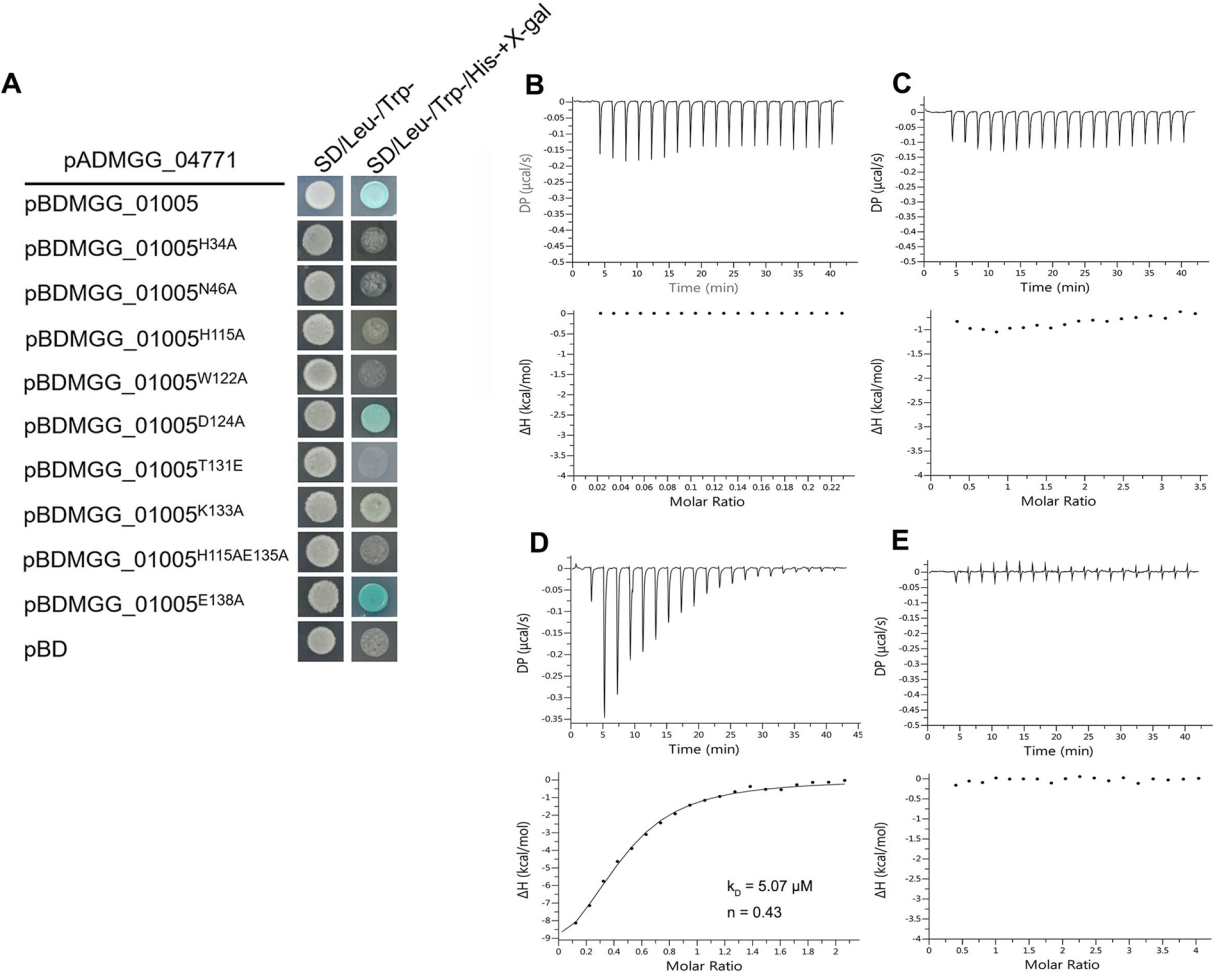
Analysis of MGG_01005 - MoDyn1I2 interactions. (**A**) Yeast two-hybrid analysis of MGG_01005- MoDyn1I2 interface mutants. (**B**–**E**) ITC thermograms for MGG_01005 mutants binding to MoDyn1I2 derived peptides. A representative experiment from three different repeats is shown. (**B**) MGG_01005^H34A^ and MoDyn1I2^117–150^, (**C**) MGG_01005^H115A^ and MoDyn1I2^117–150^, (**D**) MGG_01005^Δ(80–112)^ and MoDyn1I2^117–150^ (K_D_ = 5.07 ± 0.49 μM, n = 0.43 ± 0.018, ∆H = −12.1 kcal/mol ± 0.634 kcal/mol) and (**E**) MGG_01005 and MoDyn1I2^122–141(E132G)^.

Based on the structure of the complex, MGG_01005 residues H34, H115 and E138 together with V80 and R112 in the 80–112 loop were predicted to be important for MoDyn1I2^117–150^ binding. Therefore, the effects of mutation of these residues on the MGG_01005 - MoDyn1I2 interaction were examined by Y2H assay and ITC. The Y2H data (Fig. 4A**)**, show that Trp^+^ Leu^+^ yeast transformants expressing MoDyn1I2 and the H34A or H115A mutants of MGG_01005 are unable to grow on SD-Trp-Leu-His medium plates and show no LacZ activities indicating a loss of the interaction. However, the MGG_01005^E138A^ mutant co-expressed with MoDyn1I2 is able to grow on SD-His medium plates and has the same LacZ activity as the wild type MGG_01005. Taken together, these data demonstrate that residues H34 and H115 of MGG_01005 are essential for MoDyn1I2 binding while E138 is not.

In order to confirm these findings, H34A, H115A and Δ80–112 mutants of MGG_01005 were expressed and the binding affinity to the MoDyn1I2^117–150^ peptide measured using ITC. Consistent with the Y2H results, no interaction between mutants of MGG_01005 ^H34A^ or MGG_01005^H115A^ with the peptide was observed (Fig. 4B,C). By contrast, the deletion mutant MGG_01005 ^Δ80–112^ shows a similar binding affinity (K_D_ = 5.07 μM ± 0.49 μM) (Fig. 4D) to that of the wild type (K_D_ = 5.39 μM ± 1.09 μM) (Figure. S5E). Although both display the non-unitary stoichiometry (n = 0.43 and n = 0.55 respectively) that we attribute to the additional interactions we observe in the crystal structure. Nevertheless, taken together with the Y2H analysis these results reveal that while residues H34 and H115 are required for MoDyn1I2 binding, residues 80–112 are dispensable. To establish the requirement for E132 of MoDyn1I2 for the MGG_01005-IC binding, the interaction of a mutant MoDyn1I2^122–141^ with MGG_01005 was also analysed by ITC. These data, Fig. 4E, also reveal no detectable binding, reaffirming the importance of this key residue for MGG_01005-MoDyn1I2 interaction.

### Comparison with *Dm* dynlt1/3–dynein intermediate chain complex

**Figure 5:**
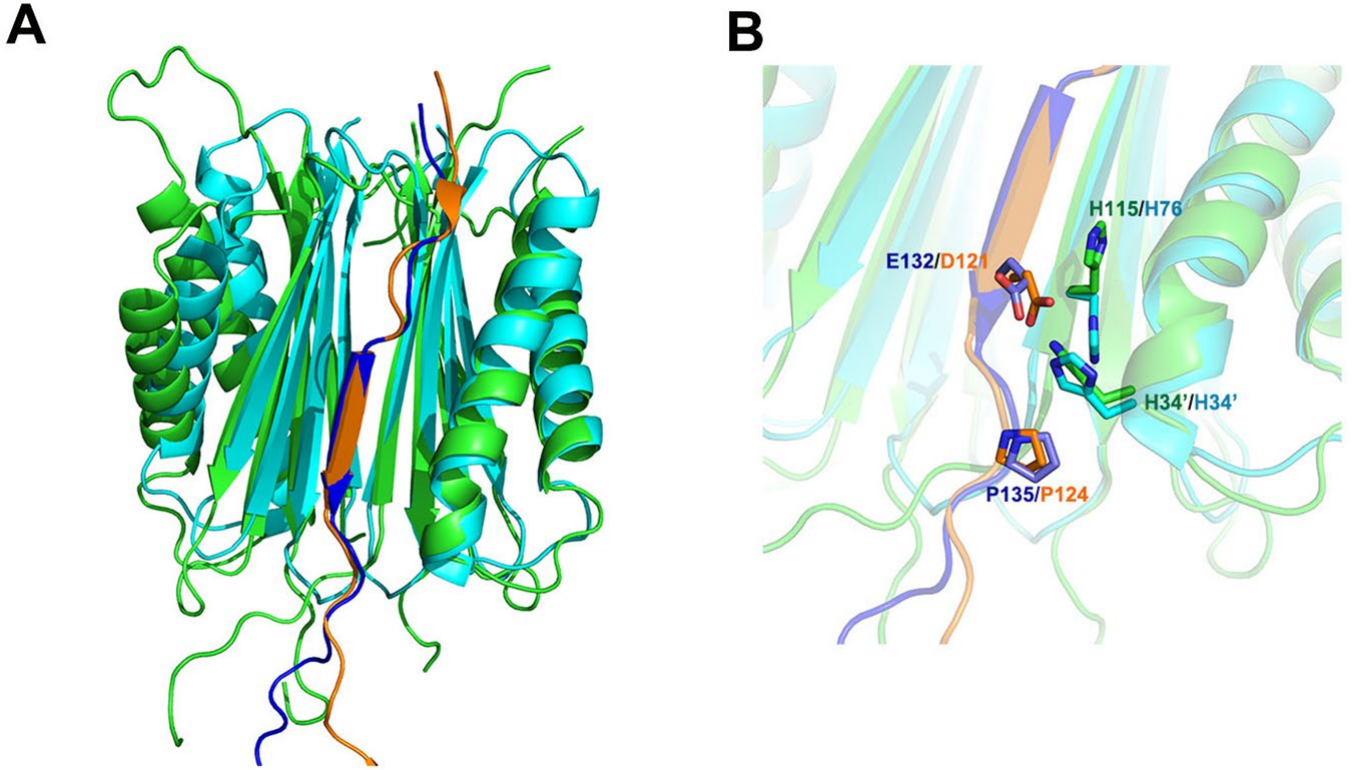
Superposition of MGG_01005-MoDyn1I2^117–150^ and *Dm* dynlt1/3-intermediate chain complex structures. (**A**) Structural alignment of the MGG_01005-MoDyn1I2^117–150^ and *Dm* dynlt1/3-intermediate chain complexes. Structures are shown in cartoon representation MGG_01005 (green), MoDyn1I2^117–150^ (blue) and *Dm* dynlt/3 (cyan), *Dm* Intermediate chain peptide (orange). (**B**) Close up view of the conserved interface. Residues that make interactions are labelled and shown in stick representation.

Although only 29 of the total 153 amino acids in MGG_01005 (less than 20%) share sequence identity with the *Dm* dynlt1/3, the structures are highly similar. Structural alignment of the *Dm* dynlt1/3-IC peptide complex (**2PG1**) with the MGG_01005- MoDyn1I2^117–150^ complex reveals an rmsd of only 1.7 Å in the Cα positions over one monomer (Fig. 5A). The interfaces with MoDyn1I2^117–150^ and the *Dm* IC peptides are also highly similar and in both complexes, hydrophobic interactions and mainchain - mainchain hydrogen bonds make major contributions (Supplementary Figure S7, Supplementary Table S3). Inspection of the structures reveals that within the bound peptides residues 128–136 for MGG_01005- MoDyn1I2^117–150^ complex and 117–125 for *Dm* dynlt1/3-IC complex have nearly identical conformation comprising a central β-strand linked by loops on both ends. More than 60% of the total hydrophobic interactions (15/25) and hydrogen bonds (11/16) are made by residues in this region of the peptide with MGG_01005. Similarly, nearly all of the interactions or contacts between *Dm* dynlt1/3 and the IC peptide are also made by residues within this region (Supplementary Figure S7, Supplementary Table S3). Among these interactions, there are two structurally conserved interactions formed by two histidine residues (H34 and H115 for MGG_01005, H34 and H76 for *Dm* dynlt1/3) with E132 (MoDyn1I2) or D121 (2PG1, the IC peptide is from *Rattus norvegicus*) (Fig. 5B). Given this interaction utilises both the conserved EXPP motif of the ICs (Fig. 2C) and the conserved di-histidine motif of dynlt1/3 it is therefore likely to be a key component of other dynlt1/3-IC complexes.

**Table 2:** Summary of Y2H and *in vitro* assays used to investigate MGG_1005 - MoDyn1I2 interactions.

MoDyn1I2 construct	MGG_1005 WT	MGG_1005 H34A	MGG_1005 H115A	MGG_1005 ∆80–112	MGG_1005 F121A	MGG_1005 T131E	MGG_1005 E133A	MGG_1005 E138A
MoDyn1I2(1–250) - Y2H	+	−	−	+	+	−	−	+
MoDyn1I2(117–150) - ITC	+	−	−	+	n.d	−	−	+
MoDyn1I2(122–141) - ITC	+	n.d	n.d	+	n.d	n.d	n.d	n.d
MoDyn1I2(122–141)E132G - ITC	−	n.d	n.d	−	n.d	n.d	n.d	n.d

Y2H, yeast-2-hybrid; ITC, isothermal titration calorimetry; n.d, no data.

Although there are strong similarities between the MGG_01005- MoDyn1I2 and *Dm* dynlt1/3-IC interfaces, two differences are apparent (Fig. 1C). One is a result of a three-residue deletion in *Dm* dynlt1/3 that corresponds to residues 136–138 (GDE) of MGG_01005 (Supplementary Figure S4A,B). In the MGG_01005-MoDyn1I2 complex, E138 makes a salt bridge with K139 of MoDyn1I2, an interaction that is unavailable in the *Dm* dynlt1/3-IC complex. However, our Y2H analysis reveals that a E138A mutation still supports interaction with MGG_01005 (Fig. 4A). The other involves interactions mediated by residues in the 80–112 loop of MGG_01005 that is absent in *Dm* dynlt1/3. However, it appears that contacts made by these residues are also not essential for the MoDyn1I2 interaction as deletion of this region still supports *w.t*. binding of MoDyn1I2^117–150^ peptide, measured by ITC (Fig. 4E). Therefore, taken together our data suggest that these less conserved regions within the dynlt1/3 family are likely not important for interaction with dynein intermediate chains (DICs).

## Discussion

### MGG_01005 is a dynein light chain, MoDynlt1/3

Structure based function-annotation has been proposed as a method that informs on structural, biochemical and biophysical properties in order to assign the cellular and molecular function of hypothetic and uncharacterised proteins that result from genome sequencing projects^10,20–22^. Based on this notion, and to test the utility we applied structure-based function-annotation to analyse the hypothetic protein, MGG_01005 in the rice blast fungus *M. oryzae*.

We first determined the crystal structure of MGG_01005 and conducted a search of the PDB using the SSM algorithm to look for proteins with the same fold. This analysis revealed matches with dynlt1/3 dynein light chains (DLCs) of *D. melanogaster* and *C. reinhardtii* strongly suggesting that MGG_01005 is the *M. oryzae* orthologue. Cytoplasmic dynein is a microtubule-based complex involved in a variety of cellular activities^23–25^. It comprises two heavy chains (DHCs) and two or three intermediate chains (DICs), light intermediate chains (DLICs) as well as DLCs^26–29^. The DLCs are the major cargo binding subunits and dynlt1/3^30^, LC8 and LC7/ Roadblock^28^ associate with the DHC through interactions with the N-terminal region of DICs to form the dynein motor complex^31,32^. Additionally, the DIC scaffold binds to other dynein components through a C-terminal WD domain. Previous studies reported that the orthologues of MGG_01005 from *S. pombe* or *U. maydis* co-located along microtubules with DHCs^33^ suggesting they were dynein light chains. Therefore, to test our hypothesis that MGG_01005 is the *M. oryzae* dynlt1/3 orthologue we assessed the capacity of MGG_01005 to bind to peptides derived from DICs. These yeast two hybrid and ITC experiments clearly demonstrated that MGG_01005 binds to the N-terminal region (1–143) of the *M. oryzae* DIC, MoDyn1I2 that contains the DLC binding motif (Table 2). We also determined the crystal structure of MGG_01005 bound to a MoDyn1I2 derived peptide (residues 117–150). Inspection of this structure and comparison with that of *Dm* dynlt1-IC peptide complexes revealed structural conservation of the interface where a di-histidine motif H34 and H115, which are conserved as H34 and H76 in *Dm* dynlt1/3, make interactions with the IC peptides. Taken together this combined structural and biochemical approach define MGG_01005 as the dynlt1/3 orthologue of *M. oryzae*. Moreover, comparison with dynlt1/3 from other species, reveals the amino acid sequence of MGG_01005 is highly conserved with that of fungi but less conserved with human, yeast and mammals (Supplementary Figure S4). Therefore, our data provides evidence for the function of annotated dynlt1/3 s in other fungi and demonstrates the power of the approach of structure-based function-annotation for the assignment of function to uncharacterised and hypothetic proteins.

## Methods

### Expression and purification of wild type and mutant MGG_01005

DNA fragments encoding *MGG_01005* were amplified by PCR from plasmid templates and inserted into a pETM13 vector, between the *NcoI* and *XhoI* restriction sites. Point or deletion mutations were made according to the manual of the Fast Mutagenesis System (Beijing TransGen Biotech Co., Ltd). pETM13 MGG_01005 constructs were transformed into *Escherichia coli* BL21(DE3) and after growth in Luria broth to an OD of 0.6 protein expression was induced by addition of 0.1 mM IPTG and the cells cultured at 18 °C for a further 16 hours. Cells were harvested by centrifugation, resuspended in 20 mM Tris-HCl, 500 mM NaCl, 1 mM DTT, pH 8.0 and lysed by sonication using a Digital Sonifier 250, Branson Co. Proteins were purified by Ni-Chelating Sepharose^TM^ Fast Flow, followed by ion exchange on Resource^TM^ Q and gel filtration on Superdex^TM^ 75 10/300 GL (GE Healthcare Co.) equilibrated in 20 mM Tris-HCl, 150 mM NaCl, pH 8.0. Purified proteins were concentrated to 8 mg/mL using an Amicon Ultra-15 centrifugal filter, flash frozen and stored at −20 °C. Selenium was incorporated into MGG_01005 by expression in the *E. coli* B834(DE3) methionine auxotroph using a defined media (Molecular dimensions) with seleno-methionine (Se-Met) substituted for methionine. The Se-Met protein and other mutants of Modynlt1/3 were purified using the same procedure as for wild type protein. Peptides derived from MoDyn1I2 (*MGG_04771*) for MGG_01005 binding studies were synthesized and HPLC purified by the Beijing Xuheyuan BioScience Company ltd. (Beijing, China). Protein and peptide concentrations were determined from the UV absorbance at 280 nm. The MGG_01005-MoDyn1I2^(117–150)^ peptide complex was prepared by mixing Modynlt1/3 and the MoDyn1I2^(117–150)^ peptide at a molar ratio of 1:1.2 followed by incubation at 4 °C for 6 h.

### Crystallization and structure determination

MGG_01005 and the MGG_01005-MoDyn1I2^(117–150)^ complex were crystalized by sitting drop vapour diffusion using an Oryx4 crystallization robot (Douglas instruments Ltd). For initial trials, a mixture of 0.25 µL protein (8 mg/mL) and an equal volume of reservoir solution was equilibrated against 33 µL of reservoir solution at either 8 °C or 16 °C. Crystals of Modynlt1/3 appeared after two or three weeks from a reservoir condition of 2.0 M ammonium citrate. After optimization, the best crystals were obtained from drops containing 0.2 µL of protein (8 mg/mL) in 20 mM Tris-HCl (pH 8.0), 150 mM NaCl and 0.2 µL 1.8 M ammonium citrate equilibrated at 16 °C. Crystals of selenium-derivatised Modynlt1/3 were obtained in the similar conditions as that of the wild type. For X-ray diffraction experiments, crystals were transferred to a reservoir solution containing 15–20% (v/v) glycerol as cryo-protectant, flash-cooled by plunging into liquid nitrogen and stored for later use.

Modynlt1/3-IC^117–150^ complex crystals were initially obtained from a solution of 2.4 M ammonium sulphate, 0.1 M Bis-Tris (pH 5.5). After varying the concentration of protein and ammonium sulphate, and optimisation of the pH and the crystallisation temperature, larger and better-looking crystals were obtained from a solution of 2.0 M ammonium sulphate, 0.1 M Bis-Tris (pH 5.2) at 8 °C.

Native and Selenium data were collected at the Shanghai Synchrotron Radiation Facility (SSRF) and processed with HKL-3000^34^. The data collection statistics are summarised in Table 1. The MGG_01005 structure was solved by single-wavelength anomalous dispersion (SAD) using the Autosol routine of PHENIX^35^. The MGG_01005-MoDyn1I2^(117–150)^ complex structure was solved by molecular replacement using the apo structure as a search model in Phaser^36^. In both cases models were subsequently improved by manual rebuilding in Coot^37^ and further refined using PHENIX with TLS (Translation/Libration/Screw) restraints^38^. Atomic coordinates and structure factors of MGG_01005 and MGG_01005-MoDyn1I2^(117–150)^ complex were deposited in the PDB with the accession codes 5HXL and 5HYC respectively. Stereochemical validation of the model was performed with MolProbity^39^. The refinement statistics and model quality parameters are detailed in Table 1. Dimer interface analysis was performed with the PISA server, (http://www.ebi.ac.uk/msd-srv/prot_int/pistart.html). Sequence alignment was performed with Clustal^40^. Figures containing structures were generated with PyMOL (PyMOL Molecular Graphics system, Version 1.3 Schrodinger, LLC).

### Yeast two-hybrid assay

Yeastmaker™ transformation system-2 (Clontech, USA) was employed to generate transformants in order to analyse protein-protein interactions using a yeast two-hybrid assay. The full-length cDNA of MGG_01005 and mutant alleles, MGG_01005^H34A^, MGG_01005^N46A^, MGG_01005^H115A^, MGG_01005^W122A^, MGG_01005^D124A^, MGG_01005^T131E^, MGG_01005^K133A^, MGG_01005^H115AE135A^, and MGG_01005^E138A^, were inserted into the bait vector pGBKT7. The full-length cDNA of MGG_04771 (MoDyn1I2) and mutant alleles, MGG_04771^1–250^, MGG_04771^1–125^, MGG_04771^126–250^, MGG_04771^141–250^, MGG_04771^251–480^, MGG_04771^481–696^, MGG_04771^251–696^, MGG_04771^1–250/P134S^, MGG_04771^1–250/F143A^, and MGG_04771^1–250/Y144A^, were inserted into the prey vector pGADKT7. Candidate bait and prey vector pairs were co-transformed into yeast strain AH109. Leu^+^ Trp^+^ transformants were isolated and then assayed for growth on SD-Leu-Trp-His media containing X-gal to evaluate protein-protein interactions.

### Isothermal titration calorimetry

Calorimetric binding experiments were carried out using an ITC_200_ microcalorimeter (MicroCal, Malvern, U.K.). The protein concentration in the cell was 60 μM and peptide ligands were at 1 mM in the syringe. The experimental temperature was maintained at 25 °C. For control experiments, peptides were titrated into a cell that contained buffer alone (20 mM Tris-HCl, 150 mM NaCl pH 8.0). For each experiment, 19 automated injections of 2 μL each were performed (duration 0.8 s) with 300 s intervals between each injection with a stirring speed of 750 rpm. Calorimetric data were recorded and fitted using the single-site binding model by allowing both n and K_A_ to float (Origin software).

### Analytical ultracentrifugation

Sedimentation velocity experiments were performed in a Beckman Optima Xl-I analytical ultracentrifuge using conventional aluminium double sector centrepieces and sapphire windows. Solvent density and the protein partial specific volumes were determined as described^41^. Prior to centrifugation, samples were prepared by exhaustive dialysis against the buffer blank solution, 20 mM Tris-HCl, 150 mM NaCl pH 8.0. Centrifugation was performed at 40,000 rpm and 277 K in an An50-Ti rotor. Interference data were acquired at time intervals of 180 s at varying Modynlt1/3 concentration (0.5–2.0 mg/ml). Data recorded from moving boundaries was analysed in terms of the size distribution function C(S) using the program SEDFIT^42–44^.

### Affinity purification and co-immunoprecipitation assays

To identify MGG_01005 co-purifying proteins, a construct expressing an MGG_01005–3XFLAG fusion protein was first generated by inserting the entire *MGG_01005* ORF into pHZ126. pHZ126-MGG_01005 was then transformed into an *M. oryzae* MGG_01005 deletion strain^11^ originally generated from the wild type *M. oryzae* strain P131 and a transformant, PDTC1, expressing MGG_01005-3XFLAG was identified by western blot analysis. For co-immunoprecipitation experiments total protein was isolated from the mycelia of the transformant PDTC1 as previously described^45,46^ and incubated with the anti-FLAG M2 Affinity Gel beads (Sigma, USA) for 8 hours on a rotary shaker at 4 °C. After washing with TBS (50 mM Tris HCl, 150 mM NaCl, pH 7.4) bound proteins were eluted with 0.1% RapiGest SF Surfactant (Waters, USA) and then digested by Trypsin (Roche, USA). The tryptic peptides were analysed by Nanoflow liquid chromatography-tandem mass spectrometry on a high-resolution hybrid linear ion trap orbitrap mass spectrometer (LTQ-Orbitrap XL, ThermoFisher, USA) coupled to an Agilent Nanoflow LC system. The tandem mass spectrometry data were queried against the NCBI nr *M. oryzae* protein database^47^. Three biological replicates were performed.

## Electronic supplementary material

Supplementary Information

Full wwPDB X-ray Structure Validation Report - 5HXL

Full wwPDB X-ray Structure Validation Report - 5HYC
