# Supplementary material for: Structure based function-annotation of hypothetical protein MGG_01005 from Magnaporthe oryzae reveals it is the dynein light chain orthologue of dynlt1/3
Source: Sci Rep. 2018 Mar 2;8:3952. doi: 10.1038/s41598-018-21667-5 (PMC5834530; doi:10.1038/s41598-018-21667-5)
Supplement: Supplementary file 1 — Supplementary Information [file 41598_2018_21667_MOESM1_ESM.pdf]

SUPPLEMENTAL MATERIAL

Structure based function-annotation of hypothetical protein MGG_01005 from *Magnaporthe oryzae* reveals it is the dynein light chain orthologue of dynlt1/3.

Guorui Li[a,c,‡], Jinguang Huang[a,b,d,‡], Jun Yang[a,b,‡], Dan He[a,b], Chao wang[a], Xiaoxuan Qi[a], Ian A. Taylor[e,*], Junfeng liu[a,*] and You-Liang Peng[a,b,*]

[a]MOA Key Laboratory of Plant Pathology, China Agricultural University, No2 Yunamingyuanxilu, Beijing, 100193, China. [b]State key Laboratory of Agrobiotechnology, China Agricultural University, No2 Yunamingyuanxilu, Beijing, 100193, China. [c]College of life science, Inner Mongolia University for Nationalities, No. 996 Xilamulun Street, Tongliao, 028043, China. [d]College of Agronomy and Plant Protection, Qingdao Agricultural University, Qingdao, Shandong, 266109, China. [e]Macromolecular Structure Laboratory, The Francis Crick Institute, London, NW1 1AT, UK.

[‡]Co-first authors, *Corresponding authors, requests for materials should be addressed to Y.P. (e-mail: pengyl@cau.edu.cn)

Supplementary Figure S1

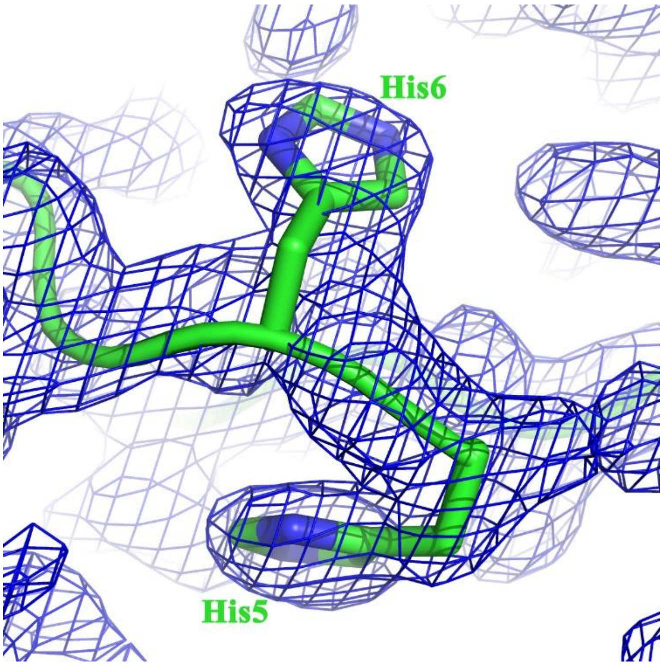

Supplementary Figure S2

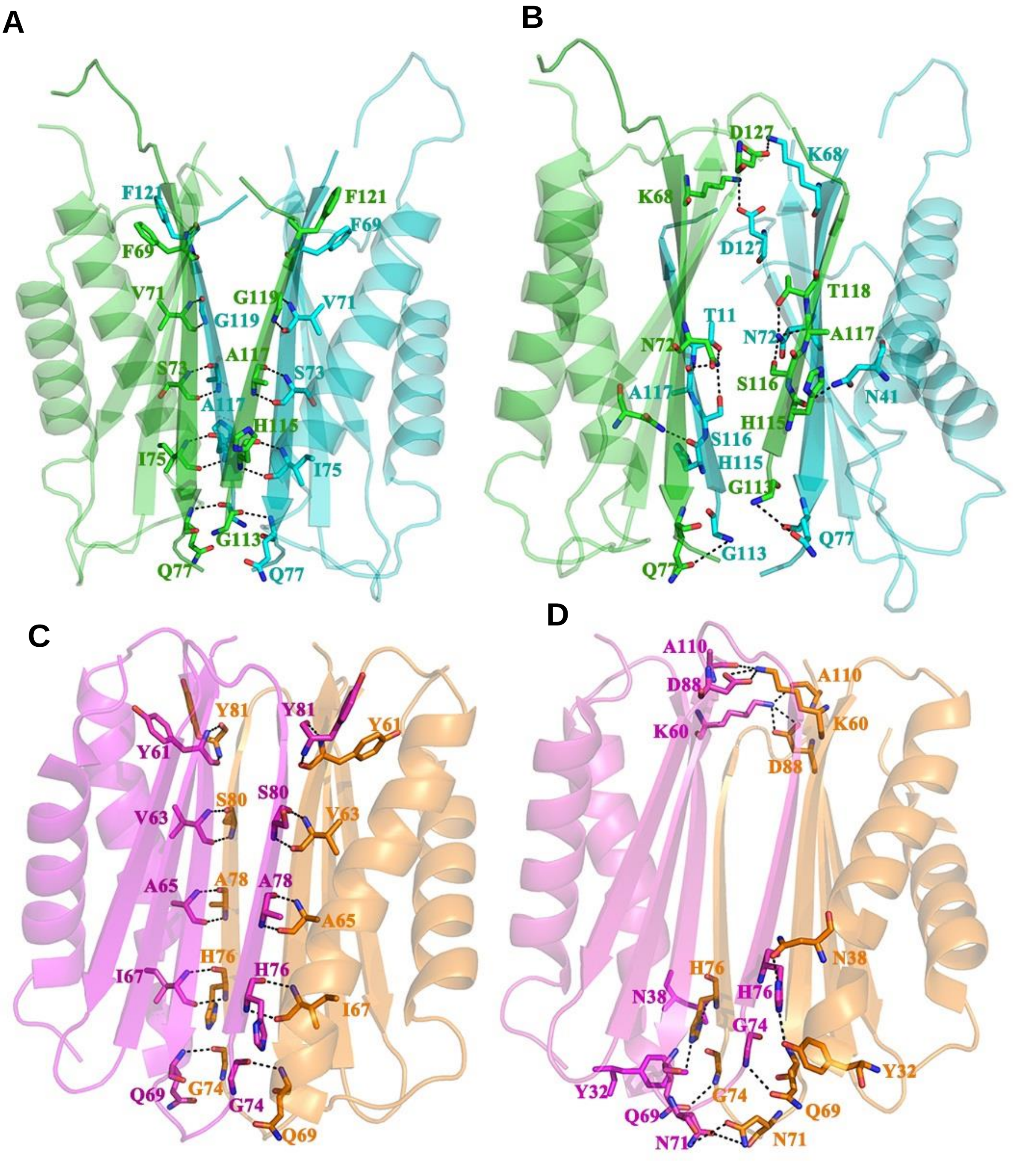

Supplementary Figure S3

A

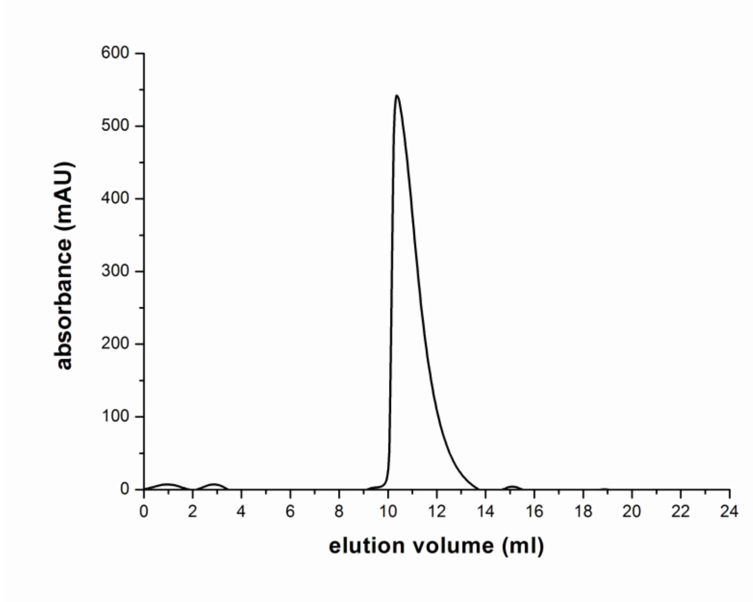

B

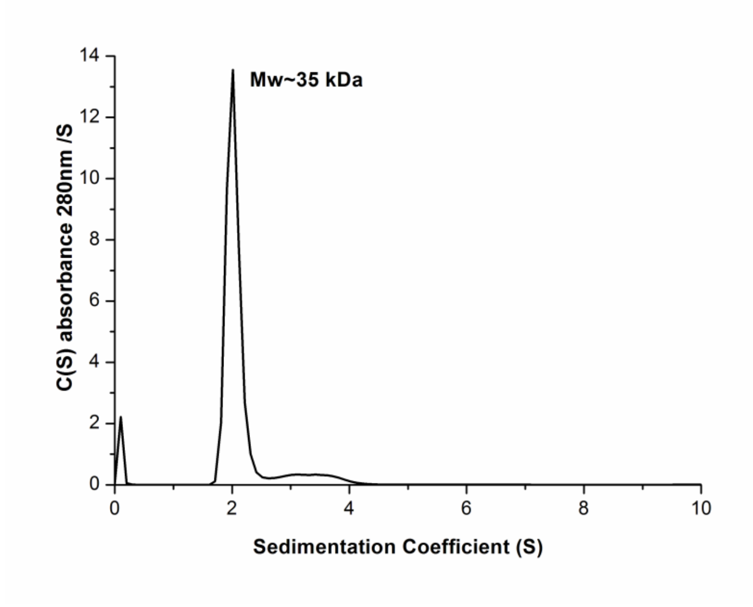

Supplementary Figure S4

A

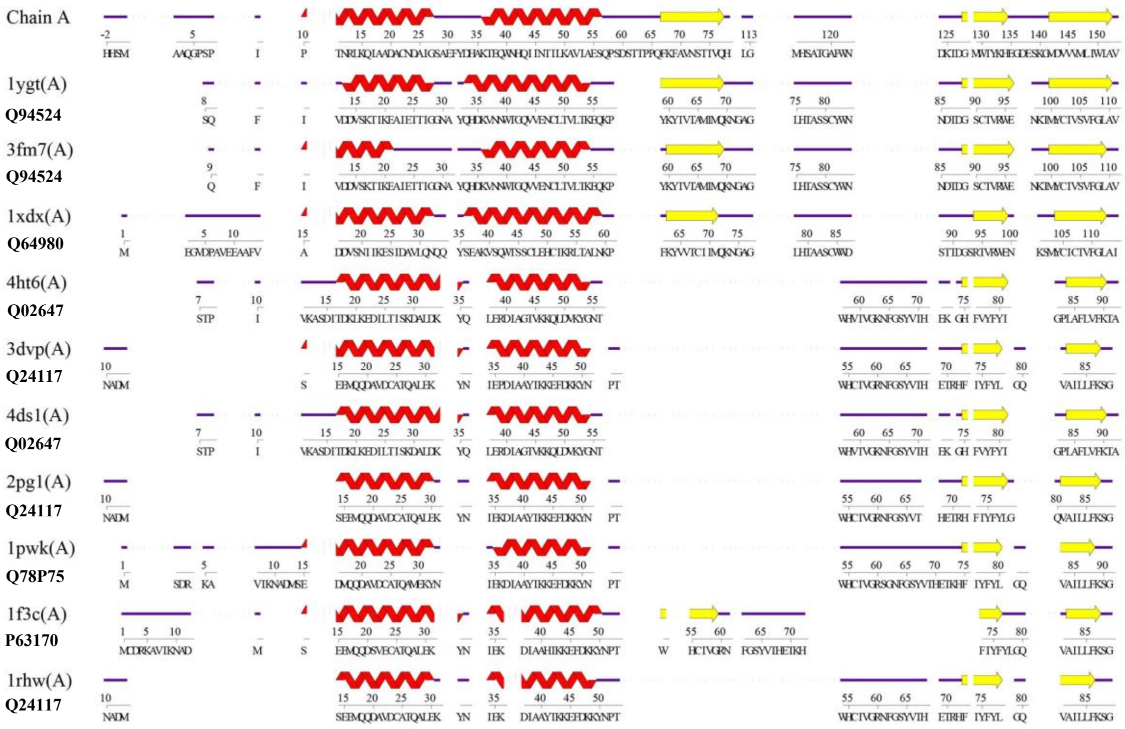

B

```
G4NCW2_MAGO7    1   --MAAQGP--SPIPTNRLKQIAADACNDAIGSAEFYDHAKTEQWNHQIINTILKAVIAESQPSDST
J9MRE3_FUSO4    1   --MATP-----PIPANRLKQIATDACNSAIGSAEFYDHAKTEQWNSTIISSVLKAVISESTP-EGA
M7UZ75_BOTF1    1   --MATP------------------ACQNALGSTEFYEHSKTESWNTTIINSILKSLISESTP-STG
Q9UTS6_SCHPO    1   --MSCP------IDSKKLEEICLEAAQPVL-KASEYDGDKTAEMNQSVIYAVLNALNKETQS----
P51807_MOUSE    1   MEDFQ-ASEETAFVVDEVSSIVKEAIESAIGG-NAYQHSKVNQWTTNVLEQTLSQLTKLGR-----
P63172_HUMAN    1   MEDYQ-AAEETAFVVDEVSNIVKEAIESAIGG-NAYQHSKVNQWTTNVVEQTLSQLTKLGK-----
Q94524_DROME    1   MDD---SREESQFIVDDVSKTIKEAIETTIGG-NAYQHDKVNNWTGQVVENCLTVLTKEQK-----
P51808_HUMAN    1   MEEYHRHCDEVGFNAEEAHNIVKECVDGVLGG-EDYNHNNINQWTASIVEQSLTHLVKLGK-----

G4NCW2_MAGO7    63  TPPQFKFAVNSTIVQH-LVPSSKLNKPAGG----ADD----------KPASTATSTDGKPHVGRRG
J9MRE3_FUSO4    59  SAPSFKFACNSTIVQH-LVPTSALNKPRGGTEIKAEEP-------HISTSSEATATDGKPHVGRRG
M7UZ75_BOTF1    46  SSPSFKYAVNSTIIQH-LVPTSSLNKSKASPPTTTEETTDDAKVTTSDAQGDAKSTDGKPHVGRRG
Q9UTS6_SCHPO    54  ----YKWIVSSTLVQKLPEDHP-----------------------------------------SRG
P51807_MOUSE    60  ---PFKYIVTCVIMQK---------------------------------------------N-GAG
P63172_HUMAN    60  ---PFKYIVTCVIMQK---------------------------------------------N-GAG
Q94524_DROME    59  ---PYKYIVTAMIMQK---------------------------------------------N-GAG
P51808_HUMAN    61  ---AYKYIVTCAVVQK---------------------------------------------S-AYG

G4NCW2_MAGO7    114 MHSATGAFWNDKTDGMWTYKHEGDESKGMDVVVMLIWIAV--
J9MRE3_FUSO4    117 MHSATGAYWDEKKDGMWTFKYDGGEGKGLDVVVMLIWVAI--
M7UZ75_BOTF1    111 MHSATGAYWNEKTDGMWSFKYEGGENKGLDVVISVIWIAL--
Q9UTS6_SCHPO    75  VHAAHAACWNCEKDGMTTIKESG---EAIDVVLSIMWISI--
P51807_MOUSE    77  LHSASSCFWDSSTDGSCTVRWEN---KTMYCIVSTFGLSI--
P63172_HUMAN    77  LHTASSCFWDSSTDGSCTVRWEN---KTMYCIVSAFGLSI--
Q94524_DROME    75  LHTASSCYWNNDTDGSCTVRWEN---KTMYCIVSVFGLAV--
P51808_HUMAN    78  FHTASSCFWDTTSDGTCTVRWEN---RTMNCIVNVFAIAIVL
```

Supplementary Figure S5

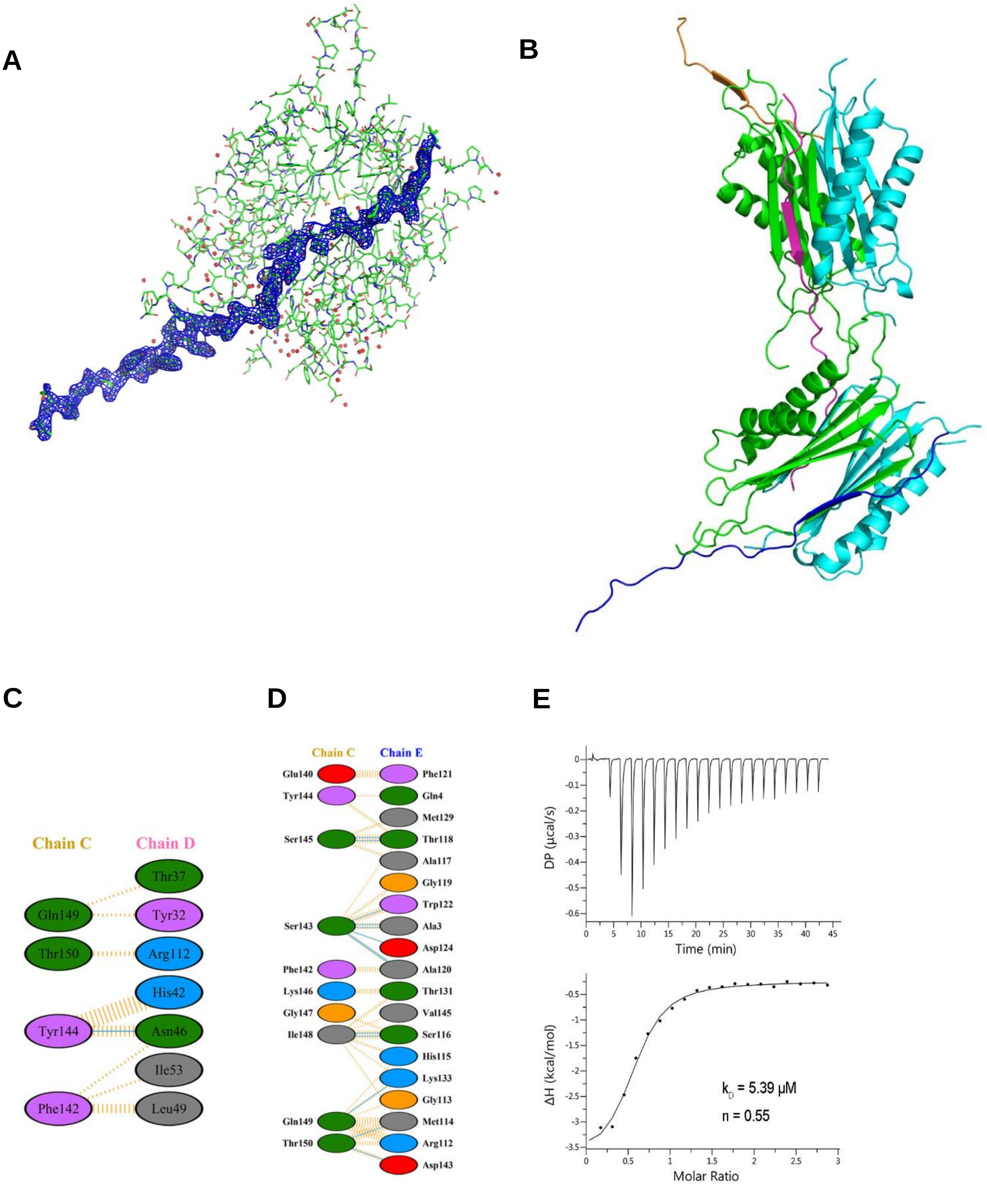

Supplementary Figure S6

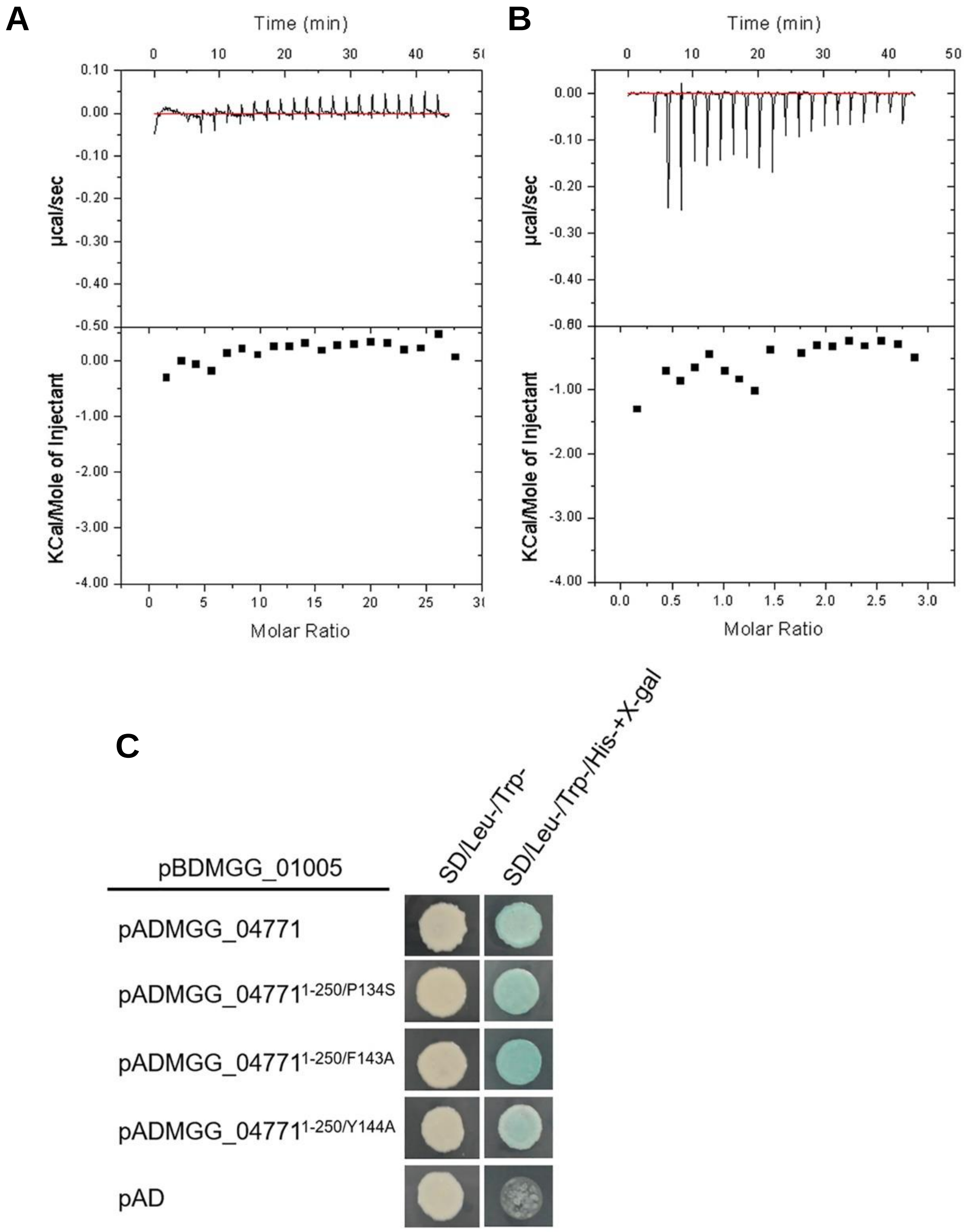

Supplementary Figure S7

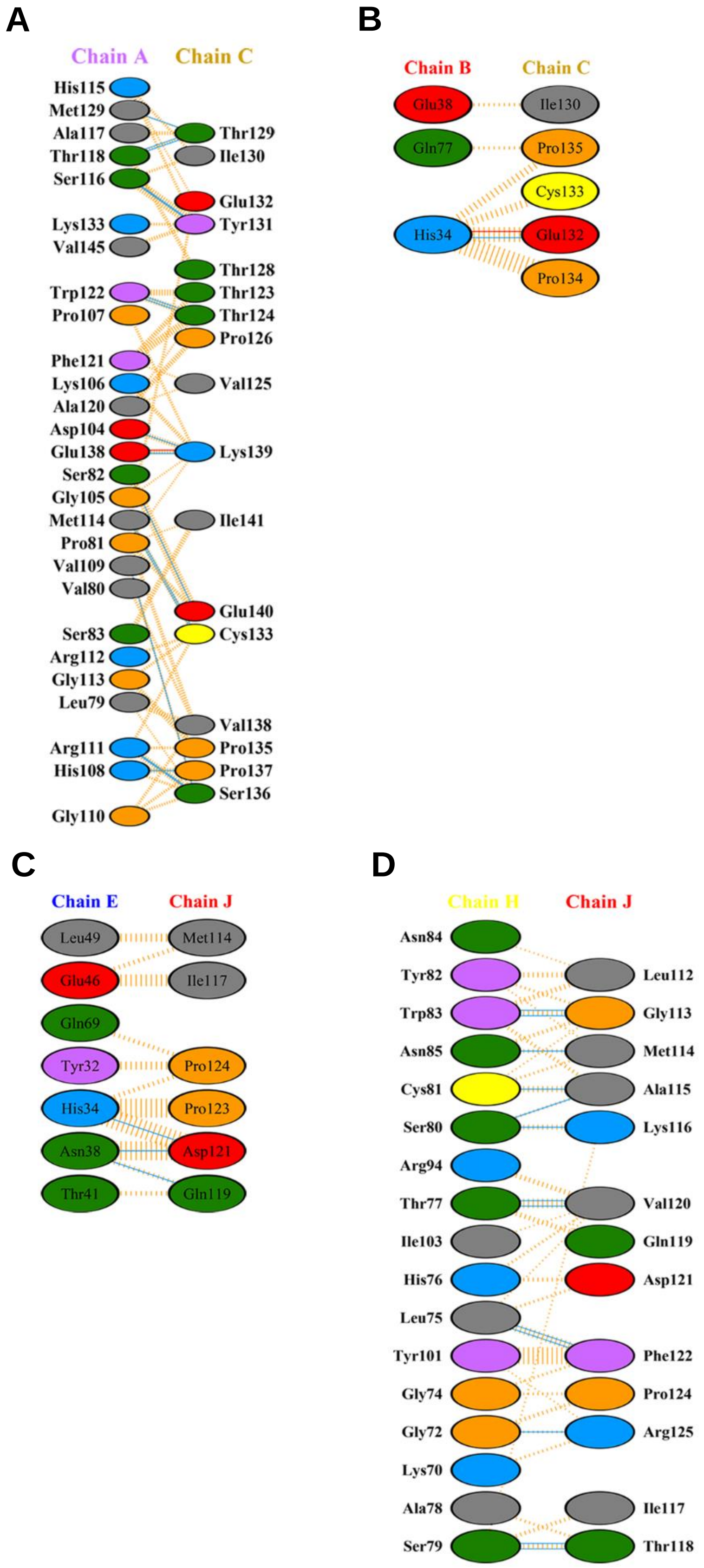

Supplementary Table S1. SSM, structural comparison search hits

Hit	Q-score	Z-score	No. SSE	RMSD (Å)	Sequence identity (full length)	PDB entry	Name/Descriptor
1	0.609	8.6	5	1.29	19.5%	1ygtA	Dynein light chain Tctex-1.
2	0.528	7.2	5	1.77	19.5%	3fm7A	Quaternary structure of Drosophila melanogaster IC/Tctex-1/LC8; Allosteric interactions of dynein light chains with dynein intermediate chain.
3	0.393	5.9	5	2.33	17.9%	1xdxA	Solution structure of the Tctex1 light chain from Chlamydomonas inner dynein arm I1.
4	0.306	4.9	4	2.06	7.2%	4ht6A	The structure of a yeast dynein Dyn2-Pac11 complex and effect on single molecule dynein motor activity.
5	0.302	4.3	4	2.23	7.9%	3dvpA	Pak1 peptide bound LC8.
6	0.297	4.7	4	2.15	7.2%	4ds1A	The structure of a yeast Dyn2-Nup159 complex and the molecular basis for the dynein light chain - nuclear pore interaction.
7	0.296	4.8	4	2.38	7.8%	2pg1A	Structural analysis of a cytoplasmic dynein light chain- intermediate chain complex.
8	0.294	3.8	4	2.19	7.8%	1pwkA	Structure of the monomeric 8-kDa dynein light chain and mechanism of domain swapped dimer assembly

Supplementary Table S2. Proteins identified by Co-immunoprecipitation-MS analysis.

No. of peptides	Protein accession	Protein score	Protein description
1	MGG_01005T0	2712	dynein light chain Tctex1 (154 aa)
2	MGG_03075T0	67	dynein light intermediate chain (562 aa)
3	MGG_07175T0	2020	KOW motif containing protein (404 aa)
4	MGG_04719T0	1256	guanine nucleotide-binding protein subunit beta-like protein (317 aa)
5	MGG_07171T0	89	hypothetical protein (398 aa)
6	MGG_03064T0	46	Rho1 guanine nucleotide exchange factor 1 (1282 aa)
7	MGG_05201T0	25	guanine nucleotide-binding protein subunit beta (347 aa)
8	MGG_06065T0	50	hsp88-like protein (713 aa)
9	MGG_04771T0	134	cytoplasmic dynein 1 intermediate chain 2 (696 aa)
10	MGG_07176T0	365	GTP-binding protein rhoA (194 aa)
11	MGG_04505T0	127	hypothetical protein (401 aa)
12	MGG_06962T0	329	GTP-binding protein ypt1 (203 aa)

Supplementary Table S3. MGG_01005 - MoDyn1I2 intermolecular interactions

Interactions MGG_01005 Chain A and MoDyn1I2 Chain C

Hydrogen bonds

	MGG_01005 Chain A				MoDyn1I2 Chain C				
	Atom No.	Atom Type	Residue Name	Residue No.	Atom No.	Atom Type	Residue Name	Residue No.	Distance (Å)
1	593	N	SER	82	2068	OE1	GLU	140	2.88
2	613	O	ASP	104	2060	NZ	LYS	139	3.08
3	650	NE2	HIS	108	2041	O	PRO	137	2.6
4	651	O	VAL	109	2037	OG	SER	136	3.34
5	663	N	ARG	111	2035	O	SER	136	3.15
6	662	O	ARG	111	2032	N	SER	136	2.79
7	688	N	MET	114	2015	O	CYS	133	3.02
8	691	O	MET	114	2012	N	CYS	133	2.92
9	706	N	SER	116	1994	O	TYR	131	2.75
10	709	O	SER	116	1991	N	TYR	131	2.54
11	717	N	THR	118	1979	O	THR	129	3.05
12	720	O	THR	118	1976	N	THR	129	3.24
13	744	N	TRP	122	1943	O	THR	124	2.82
14	747	O	TRP	122	1940	N	THR	124	2.7
15	808	SD	MET	129	1981	OG1	THR	129	3.17
16	890	OE1	GLU	138	2060	NZ	LYS	139	3.06

Salt bridges

	Chain A				Chain C				
	Atom No.	Atom Type	Residue Name	Residue No.	Atom No.	Atom Type	Residue Name	Residue No.	Distance (Å)
1	890	OE1	GLU	138	2060	NZ	LYS	139	3.06

Hydrophobic interactions

Hydrophobic interactions, Chain A and Chain C: 134

Interactions MGG_01005 Chain B and MoDyn1I2 Chain C

Hydrogen bonds

	MGG_01005 Chain B				MoDyn1I2 Chain C				
	Atom No.	Atom Type	Residue Name	Residue No.	Atom No.	Atom Type	Residue Name	Residue No.	Distance (Å)
1	1242	NE2	HIS	34	2011	OE2	GLU	132	2.34

Salt bridges

	Chain B				Chain C				
	Atom No.	Atom Type	Residue Name	Residue No.	Atom No.	Atom Type	Residue name	Residue No.	Distance (Å)
1	1242	NE2	HIS	34	2011	OE2	GLU	132	2.34

Hydrophobic interactions

Hydrophobic interactions, Chain B and Chain C:22

Supplementary Figure Legends

Figure S1. Experimental electron density. A 2Fo-Fc electron density map contoured at 1.0 σ of the region around the N-terminal His-tag. The map is shown as blue mesh and histidine residues built into the density are shown in stick representation.

Figure S2. MGG_01005 and *Dm* dynlt1/3 homodimer interfaces. (**A**, **B**) Interactions between Chain A (green) and Chain B (cyan) of the MGG_01005 homodimer, (**A**) mainchain - mainchain interactions, (**B**) side chain - side chain interactions. (**C**, **D**) Interactions at the homodimer interface of *Dm* dynlt1/3 (PDB ID: 2pg1), Chain F (magenta) and Chain J (orange) (**C**) Mainchain - mainchain interactions (**D**) side chain - side chain interactions. In panels **A-D** hydrogen bond interactions are shown as black dashed lines.

Figure S3. Solution molecular mass analysis of MGG_01005

(**A**) Gel filtration chromatography analysis of the MGG_01005. A sample of MGG_01005 (50µl @ 5 mg/mL) was applied to a Superdex 75 10/30 column. MGG_01005 elutes as a single peak with an elution volume consistent with a dimer molecular mass. (**B**) Sedimentation velocity analytical ultracentrifugation (SV-AUC) analysis of MGG_01005. The best fit continuous distribution of sedimentation coefficients function [C(S)] derived from SV-AUC data recorded from MGG_01005 at 0.5 mg/mL. Inset, the molar mass derived from the peak and best fit frictional ratio to the C(S) function.

Figure S4. Protein sequence alignments.

(**A**) Secondary structure based sequence alignments of MGG_01005 with dynlt1/3 orthologues. The positions of helices (red) and β-strands (yellow) are shown above the alignment. The upper reference sequence is from Chain A of MGG_01005. For the other

sequences the PDB and Uniprot accession codes for each protein are indicated (left). 1ygt and 3fm7, Q94524, *Drosophila melanogaster*; 1xdx, Q64980, *Chlamydomonas reinhardtii*; 4ht6 and 4ds1, Q02647, *Saccharomyces cerevisiae*; 3dvp and 2pg1, Q24117, *Drosophila melanogaster*; 1pwk, Q78p75, *Rattus norvegicus*; 1f3c, Q63170, *Rattus norvegicus*. (**B**) Primary sequence alignments of MGG_01005 with dynlt1/3 orthologues. Uniprot number and Species are shown (left). Highly conserved residues are coloured red and boxed.

Figure S5. MGG_01005-MoDyn1I2$^{117\text{-}150}$ interactions at the second interface. (A) A 2Fo-Fc electron density map of the MoDyn1I2$^{117\text{-}150}$ peptide within the complex (blue mesh) contoured at 1.0 σ. Residues 142-150 of the peptide extend from the canonical binding site. **(B)** View of the packing of MGG_01005-MoDyn1/2117-150 complexes in the crystal structure. Three IC peptides (blue, orange, and magenta) bind to two MGG_01005 dimers (green and cyan). (**C-D**) Schematic representation of MGG_01005-MoDyn1I2$^{117\text{-}150}$ interactions at the second interface. Chain D and E (MGG_01005) Chain C, MoDyn1I2$^{117\text{-}150}$. Residues are colour coded with respect to side chain properties. Blue, positively charged (H, K, R); red, negatively charged (D, E); green, neutral (S, T, N, Q); grey, aliphatic (A, V, L, I, M); mauve, aromatic (F, Y, W); orange, Proline & Glycine (P, G); yellow, cysteine (C). Blue lines represent intermolecular hydrogen bonds and hydrophobic interactions are shown as orange dashes. (**E**)) ITC thermogram for MGG_01005 binding to a MoDyn1I2$^{(117\text{-}150}$ peptide. A representative experiment from three different repeats is shown (K_D =5.39 μM ± 1.09 μM, n =0.55 ± 0.02, ΔH (kcal/mol) = -3.55 ± 0.194, ΔG (kcal/mol) = -7.19).

Figure S6. Interaction of MGG_01005 with MoDyn1I2. (**A, B**) ITC analysis of MGG_01005 binding to (**A**) MoDyn1I2$^{142\text{-}150}$ and (**B**) MoDyn1I2$^{141\text{-}158}$ peptides. The upper panels are raw thermograms and the lower panels the heats at each injection. A

representative experiment from three different repeats is shown. (**C**) Yeast two-hybrid analysis of the interaction between MGG_01005 and MoDyn1I2 mutants containing amino acid substitutions within the 141-150 region.

Figure S7. Schematic of MGG_01005 and *Dm* dynlt1/3 interactions with intermediate chain peptides. Residues are represented by ovals with the same colour coding as in Figure **S5**. (**A)** Interactions between of MGG_01005 (Chain A) and MoDyn1I2$^{117\text{-}150}$ (Chain C). (**B**) Interactions between of MGG_01005 (Chain B) and MoDyn1I2$^{117\text{-}150}$ (Chain C). (**C**) Interactions between of *Dm* dynlt1/3 (Chain E) and *Dm* intermediate chain (Chain J). (D) Interactions between of *Dm* dynlt1/3 (Chain H) and *Dm* intermediate chain (Chain J). Blue and red lines represent hydrogen bonds and salt bridges respectively. The hydrophobic interactions are shown as orange dashes.

Supplementary Table S1. SSM, structural comparison search hits.

Supplementary Table S2. Proteins identified by Co-immunoprecipitation-MS analysis.

Supplementary Table S3. MGG_01005 - MoDyn1I2 intermolecular interactions.
