# Supplementary material for: Structure based function-annotation of hypothetical protein MGG_01005 from Magnaporthe oryzae reveals it is the dynein light chain orthologue of dynlt1/3
Source: Sci Rep. 2018 Mar 2;8:3952. doi: 10.1038/s41598-018-21667-5 (PMC5834530; doi:10.1038/s41598-018-21667-5)
Supplement: Supplementary file 3 — Full wwPDB X-ray Structure Validation Report - 5HYC [file 41598_2018_21667_MOESM3_ESM.pdf]

Full wwPDB X-ray Structure Validation Report (i)

Feb 23, 2016 – 02:16 AM EST

PDB ID	:	5HYC
Title	:	Structure based function annotation of a hypothetical protein MGG_01005 related to the development of rice blast fungus
Authors	:	Liu, J.; Li, G.; Huang, J.; Peng, Y.-l.
Deposited on	:	2016-02-01
Resolution	:	2.40 Å(reported)

This is a Full wwPDB X-ray Structure Validation Report.
We welcome your comments at validation@mail.wwpdb.org
A user guide is available at
http://wwpdb.org/validation/2016/XrayValidationReportHelp
with specific help available everywhere you see the (i) symbol.

The following versions of software and data (see references (i)) were used in the production of this report:

MolProbity	:	4.02b-467
Mogul	:	unknown
Xtriage (Phenix)	:	1.9-1692
EDS	:	rb-20026919
Percentile statistics	:	20151230.v01 (using entries in the PDB archive December 30th 2015)
Refmac	:	5.8.0122
CCP4	:	6.1.3
Ideal geometry (proteins)	:	Engh & Huber (2001)
Ideal geometry (DNA, RNA)	:	Parkinson et al. (1996)
Validation Pipeline (wwPDB-VP)	:	rb-20026919

CONFIDENTIAL VALIDATION REPORT

1 Overall quality at a glance (i)

The following experimental techniques were used to determine the structure:
X-RAY DIFFRACTION

The reported resolution of this entry is 2.40 Å.

Percentile scores (ranging between 0-100) for global validation metrics of the entry are shown in the following graphic. The table shows the number of entries on which the scores are based.

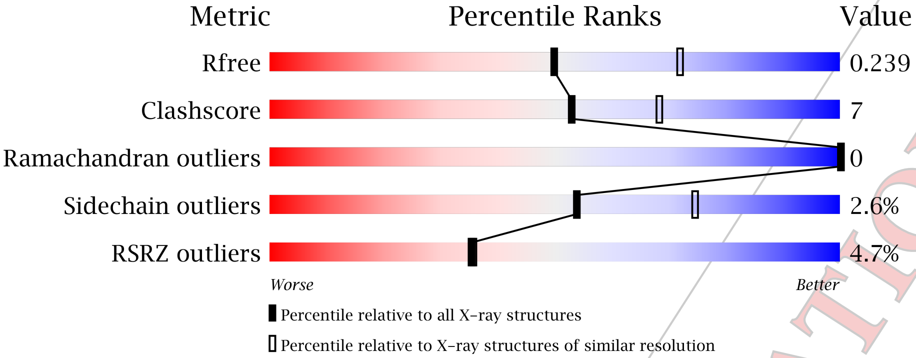

Metric	Whole archive (#Entries)	Similar resolution (#Entries, resolution range(Å))
R_{free}	91344	2919 (2.40-2.40)
Clashscore	102246	3407 (2.40-2.40)
Ramachandran outliers	100387	3351 (2.40-2.40)
Sidechain outliers	100360	3352 (2.40-2.40)
RSRZ outliers	91569	2928 (2.40-2.40)

The table below summarises the geometric issues observed across the polymeric chains and their fit to the electron density. The red, orange, yellow and green segments on the lower bar indicate the fraction of residues that contain outliers for >=3, 2, 1 and 0 types of geometric quality criteria. A grey segment represents the fraction of residues that are not modelled. The numeric value for each fraction is indicated below the corresponding segment, with a dot representing fractions <=5% The upper red bar (where present) indicates the fraction of residues that have poor fit to the electron density. The numeric value is given above the bar.

Mol	Chain	Length	Quality of chain
1	A	161	2% / 71% 9% • 19%
1	B	161	4% / 63% 9% • 27%
2	C	35	9% / 69% 11% • 17%

CONFIDENTIAL VALIDATION REPORT

2 Entry composition (i)

There are 3 unique types of molecules in this entry. The entry contains 2223 atoms, of which 0 are hydrogens and 0 are deuteriums.

In the tables below, the ZeroOcc column contains the number of atoms modelled with zero occupancy, the AltConf column contains the number of residues with at least one atom in alternate conformation and the Trace column contains the number of residues modelled with at most 2 atoms.

- Molecule 1 is a protein called Uncharacterized protein.

Mol	Chain	Residues	Atoms					ZeroOcc	AltConf	Trace
			Total	C	N	O	S			
1	A	130	1005	633	176	191	5	0	0	0
1	B	118	917	582	158	172	5	0	0	0

There are 16 discrepancies between the modelled and reference sequences:

Chain	Residue	Modelled	Actual	Comment	Reference
A	154	LEU	-	expression tag	UNP G4NCW2
A	155	GLU	-	expression tag	UNP G4NCW2
A	156	HIS	-	expression tag	UNP G4NCW2
A	157	HIS	-	expression tag	UNP G4NCW2
A	158	HIS	-	expression tag	UNP G4NCW2
A	159	HIS	-	expression tag	UNP G4NCW2
A	160	HIS	-	expression tag	UNP G4NCW2
A	161	HIS	-	expression tag	UNP G4NCW2
B	154	LEU	-	expression tag	UNP G4NCW2
B	155	GLU	-	expression tag	UNP G4NCW2
B	156	HIS	-	expression tag	UNP G4NCW2
B	157	HIS	-	expression tag	UNP G4NCW2
B	158	HIS	-	expression tag	UNP G4NCW2
B	159	HIS	-	expression tag	UNP G4NCW2
B	160	HIS	-	expression tag	UNP G4NCW2
B	161	HIS	-	expression tag	UNP G4NCW2

- Molecule 2 is a protein called Cytoplasmic dynein 1 intermediate chain 2.

Mol	Chain	Residues	Atoms					ZeroOcc	AltConf	Trace
			Total	C	N	O	S			
2	C	29	225	148	32	44	1	0	0	0

- Molecule 3 is water.

CONFIDENTIAL VALIDATION REPORT

Mol	Chain	Residues	Atoms	ZeroOcc	AltConf
3	A	28	Total O<br>28 28	0	0
3	B	44	Total O<br>44 44	0	0
3	C	4	Total O<br>4 4	0	0

CONFIDENTIAL VALIDATION REPORT

3 Residue-property plots (i)

These plots are drawn for all protein, RNA and DNA chains in the entry. The first graphic for a chain summarises the proportions of errors displayed in the second graphic. The second graphic shows the sequence view annotated by issues in geometry and electron density. Residues are color-coded according to the number of geometric quality criteria for which they contain at least one outlier: green = 0, yellow = 1, orange = 2 and red = 3 or more. A red dot above a residue indicates a poor fit to the electron density (RSRZ > 2). Stretches of 2 or more consecutive residues without any outlier are shown as a green connector. Residues present in the sample, but not in the model, are shown in grey.

• Molecule 1: Uncharacterized protein

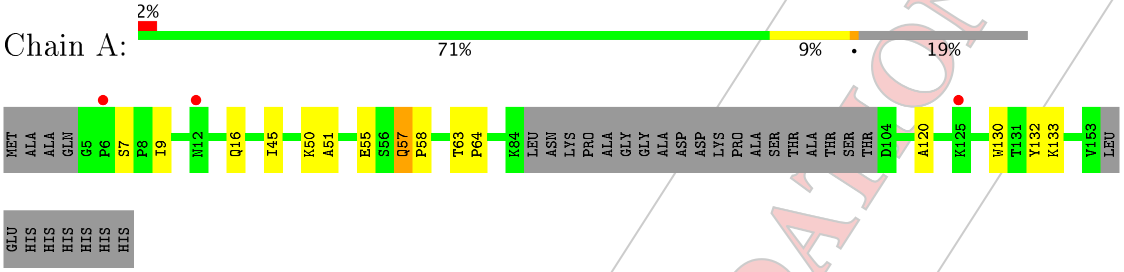

• Molecule 1: Uncharacterized protein

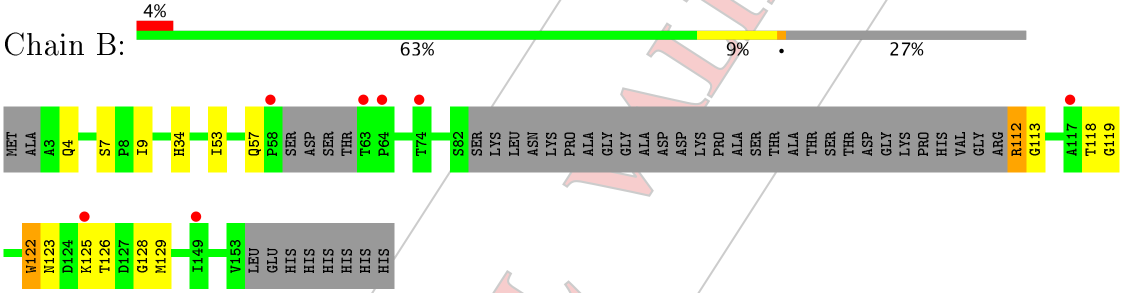

• Molecule 2: Cytoplasmic dynein 1 intermediate chain 2

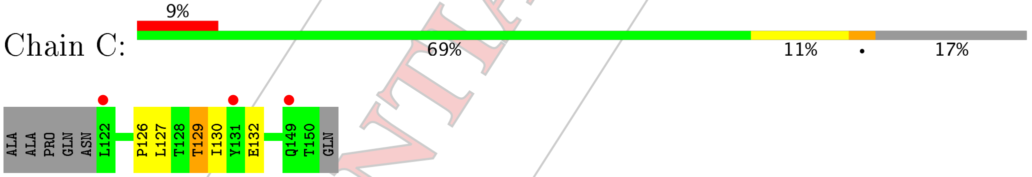

CONFIDENTIAL VALIDATION REPORT

4 Data and refinement statistics (i)

Property	Value	Source
Space group	I 41 2 2	Depositor
Cell constants<br>a, b, c, α, β, γ	105.10Å 105.10Å 166.54Å<br>90.00° 90.00° 90.00°	Depositor
Resolution (Å)	29.63 – 2.40<br>29.63 – 2.40	Depositor<br>EDS
% Data completeness<br>(in resolution range)	98.9 (29.63-2.40)<br>99.0 (29.63-2.40)	Depositor<br>EDS
R_{merge}	(Not available)	Depositor
R_{sym}	(Not available)	Depositor
$< I/\sigma(I) >$ [1]	2.83 (at 2.39Å)	Xtriage
Refinement program	PHENIX (phenix.refine: 1.9_1692)	Depositor
R, R_{free}	0.206 , 0.235<br>0.209 , 0.239	Depositor<br>DCC
R_{free} test set	1843 reflections (10.01%)	DCC
Wilson B-factor (Å^2)	48.1	Xtriage
Anisotropy	0.273	Xtriage
Bulk solvent k_{sol}(e/Å^3), B_{sol}(Å^2)	0.35 , 43.4	EDS
Estimated twinning fraction	No twinning to report.	Xtriage
L-test for twinning[2]	$< \|L\| >$ = 0.49, $< L^2 >$ = 0.33	Xtriage
Outliers	0 of 18442 reflections	Xtriage
F_o,F_c correlation	0.94	EDS
Total number of atoms	2223	wwPDB-VP
Average B, all atoms (Å^2)	55.0	wwPDB-VP

Xtriage's analysis on translational NCS is as follows: *The largest off-origin peak in the Patterson function is 5.98% of the height of the origin peak. No significant pseudotranslation is detected.*

[1] Intensities estimated from amplitudes.

[2] Theoretical values of $< |L| >$, $< L^2 >$ for acentric reflections are 0.5, 0.375 respectively for untwinned datasets, and 0.333, 0.2 for perfectly twinned datasets.

CONFIDENTIAL VALIDATION REPORT

5 Model quality (i)

5.1 Standard geometry (i)

The Z score for a bond length (or angle) is the number of standard deviations the observed value is removed from the expected value. A bond length (or angle) with $|Z| > 5$ is considered an outlier worth inspection. RMSZ is the root-mean-square of all Z scores of the bond lengths (or angles).

Mol	Chain	Bond lengths		Bond angles	
		RMSZ	#$|Z|$ >5	RMSZ	#$|Z|$ >5
1	A	0.51	0/1031	0.60	0/1401
1	B	0.45	0/940	0.56	0/1278
2	C	0.54	0/231	0.62	0/317
All	All	0.49	0/2202	0.59	0/2996

Chiral center outliers are detected by calculating the chiral volume of a chiral center and verifying if the center is modelled as a planar moiety or with the opposite hand. A planarity outlier is detected by checking planarity of atoms in a peptide group, atoms in a mainchain group or atoms of a sidechain that are expected to be planar.

Mol	Chain	#Chirality outliers	#Planarity outliers
2	C	0	1

There are no bond length outliers.

There are no bond angle outliers.

There are no chirality outliers.

All (1) planarity outliers are listed below:

Mol	Chain	Res	Type	Group
2	C	130	ILE	Peptide

5.2 Too-close contacts (i)

In the following table, the Non-H and H(model) columns list the number of non-hydrogen atoms and hydrogen atoms in the chain respectively. The H(added) column lists the number of hydrogen atoms added and optimized by MolProbity. The Clashes column lists the number of clashes within the asymmetric unit, whereas Symm-Clashes lists symmetry related clashes.

Mol	Chain	Non-H	H(model)	H(added)	Clashes	Symm-Clashes
1	A	1005	0	972	13	0
1	B	917	0	886	14	0

Continued on next page...

CONFIDENTIAL VALIDATION REPORT

Continued from previous page...

Mol	Chain	Non-H	H(model)	H(added)	Clashes	Symm-Clashes
2	C	225	0	231	4	0
3	A	28	0	0	1	1
3	B	44	0	0	1	2
3	C	4	0	0	1	0
All	All	2223	0	2089	28	2

The all-atom clashscore is defined as the number of clashes found per 1000 atoms (including hydrogen atoms). The all-atom clashscore for this structure is 7.

All (28) close contacts within the same asymmetric unit are listed below, sorted by their clash magnitude.

Atom-1	Atom-2	Interatomic distance (Å)	Clash overlap (Å)
1:A:57:GLN:HA	1:A:57:GLN:HE21	1.38	0.89
1:B:53:ILE:O	1:B:57:GLN:NE2	2.12	0.82
1:A:57:GLN:HA	1:A:57:GLN:NE2	1.99	0.76
1:B:123:ASN:OD1	1:B:125:LYS:HG2	1.88	0.73
1:A:57:GLN:NE2	1:A:58:PRO:HD2	2.06	0.71
1:A:57:GLN:HE21	1:A:58:PRO:HD2	1.59	0.68
1:B:112:ARG:NH1	3:B:201:HOH:O	2.26	0.67
1:A:120:ALA:O	2:C:126:PRO:HD2	1.96	0.65
1:B:4:GLN:HG2	1:B:122:TRP:CZ2	2.32	0.65
2:C:127:LEU:HD12	2:C:127:LEU:H	1.62	0.63
1:A:57:GLN:HE21	1:A:57:GLN:CA	2.10	0.62
1:B:7:SER:OG	1:B:9:ILE:O	2.16	0.58
1:A:7:SER:OG	1:A:9:ILE:O	2.18	0.57
1:B:122:TRP:HH2	1:B:129:MET:HG2	1.69	0.57
1:B:125:LYS:HG3	1:B:126:THR:HG23	1.86	0.57
1:B:4:GLN:HG2	1:B:122:TRP:HZ2	1.70	0.55
1:B:118:THR:HB	1:B:129:MET:HE1	1.90	0.54
1:A:50:LYS:NZ	3:A:201:HOH:O	2.00	0.53
1:B:123:ASN:HD21	1:B:125:LYS:HE3	1.74	0.52
1:B:122:TRP:CH2	1:B:128:GLY:HA2	2.48	0.48
1:A:130:TRP:CH2	1:A:132:TYR:HB2	2.50	0.46
2:C:129:THR:HG23	3:C:201:HOH:O	2.16	0.46
1:A:51:ALA:O	1:A:55:GLU:HG3	2.17	0.45
1:B:34:HIS:NE2	2:C:132:GLU:OE2	2.34	0.45
1:B:112:ARG:HB2	1:B:113:GLY:H	1.49	0.43
1:A:63:THR:HA	1:A:64:PRO:HD3	1.81	0.42
1:A:45:ILE:HG21	1:B:119:GLY:HA3	2.00	0.42
1:A:57:GLN:HE21	1:A:58:PRO:CD	2.32	0.40

CONFIDENTIAL VALIDATION REPORT

All (2) symmetry-related close contacts are listed below. The label for Atom-2 includes the symmetry operator and encoded unit-cell translations to be applied.

Atom-1	Atom-2	Interatomic distance (Å)	Clash overlap (Å)
3:A:202:HOH:O	3:B:226:HOH:O[11_454]	2.06	0.14
3:B:212:HOH:O	3:B:238:HOH:O[10_555]	2.14	0.06

5.3 Torsion angles (i)

5.3.1 Protein backbone (i)

In the following table, the Percentiles column shows the percent Ramachandran outliers of the chain as a percentile score with respect to all X-ray entries followed by that with respect to entries of similar resolution.

The Analysed column shows the number of residues for which the backbone conformation was analysed, and the total number of residues.

Mol	Chain	Analysed	Favoured	Allowed	Outliers	Percentiles	
1	A	126/161 (78%)	124 (98%)	2 (2%)	0	100	100
1	B	112/161 (70%)	111 (99%)	1 (1%)	0	100	100
2	C	27/35 (77%)	27 (100%)	0	0	100	100
All	All	265/357 (74%)	262 (99%)	3 (1%)	0	100	100

There are no Ramachandran outliers to report.

5.3.2 Protein sidechains (i)

In the following table, the Percentiles column shows the percent sidechain outliers of the chain as a percentile score with respect to all X-ray entries followed by that with respect to entries of similar resolution.

The Analysed column shows the number of residues for which the sidechain conformation was analysed, and the total number of residues.

Mol	Chain	Analysed	Rotameric	Outliers	Percentiles	
1	A	109/132 (83%)	106 (97%)	3 (3%)	51	72
1	B	98/132 (74%)	96 (98%)	2 (2%)	63	81
2	C	28/32 (88%)	27 (96%)	1 (4%)	42	63
All	All	235/296 (79%)	229 (97%)	6 (3%)	54	74

CONFIDENTIAL VALIDATION REPORT

All (6) residues with a non-rotameric sidechain are listed below:

Mol	Chain	Res	Type
1	A	16	GLN
1	A	57	GLN
1	A	133	LYS
1	B	112	ARG
1	B	122	TRP
2	C	129	THR

Some sidechains can be flipped to improve hydrogen bonding and reduce clashes. All (1) such sidechains are listed below:

Mol	Chain	Res	Type
1	A	57	GLN

5.3.3 RNA (i)

There are no RNA molecules in this entry.

5.4 Non-standard residues in protein, DNA, RNA chains (i)

There are no non-standard protein/DNA/RNA residues in this entry.

5.5 Carbohydrates (i)

There are no carbohydrates in this entry.

5.6 Ligand geometry (i)

There are no ligands in this entry.

5.7 Other polymers (i)

There are no such residues in this entry.

5.8 Polymer linkage issues (i)

There are no chain breaks in this entry.

CONFIDENTIAL VALIDATION REPORT

6 Fit of model and data (i)

6.1 Protein, DNA and RNA chains (i)

In the following table, the column labelled '#RSRZ> 2' contains the number (and percentage) of RSRZ outliers, followed by percent RSRZ outliers for the chain as percentile scores relative to all X-ray entries and entries of similar resolution. The OWAB column contains the minimum, median, 95th percentile and maximum values of the occupancy-weighted average B-factor per residue. The column labelled 'Q< 0.9' lists the number of (and percentage) of residues with an average occupancy less than 0.9.

Mol	Chain	Analysed	<RSRZ>	#RSRZ>2			OWAB(Å^2)	Q<0.9
1	A	130/161 (80%)	0.18	3 (2%)	64	63	33, 45, 94, 108	0
1	B	118/161 (73%)	0.33	7 (5%)	26	26	35, 52, 104, 133	0
2	C	29/35 (82%)	0.49	3 (10%)	9	8	37, 60, 88, 90	0
All	All	277/357 (77%)	0.28	13 (4%)	35	36	33, 50, 94, 133	0

All (13) RSRZ outliers are listed below:

Mol	Chain	Res	Type	RSRZ
2	C	131	TYR	4.0
1	B	63	THR	3.9
1	B	58	PRO	3.8
1	A	125	LYS	3.5
1	B	64	PRO	3.5
2	C	122	LEU	3.3
1	A	6	PRO	2.7
1	B	149	ILE	2.5
1	B	117	ALA	2.5
1	A	12	ASN	2.3
1	B	125	LYS	2.1
2	C	149	GLN	2.1
1	B	74	THR	2.0

6.2 Non-standard residues in protein, DNA, RNA chains (i)

There are no non-standard protein/DNA/RNA residues in this entry.

6.3 Carbohydrates (i)

There are no carbohydrates in this entry.

CONFIDENTIAL VALIDATION REPORT

6.4 Ligands (i)

There are no ligands in this entry.

6.5 Other polymers (i)

There are no such residues in this entry.

CONFIDENTIAL VALIDATION REPORT
